# *Mycobacterium tuberculosis*-induced miR-155 subverts autophagy by targeting ATG3 in human dendritic cells

**DOI:** 10.1371/journal.ppat.1006790

**Published:** 2018-01-04

**Authors:** Marilena P. Etna, Alessandro Sinigaglia, Angela Grassi, Elena Giacomini, Alessandra Romagnoli, Manuela Pardini, Martina Severa, Melania Cruciani, Fabiana Rizzo, Eleni Anastasiadou, Barbara Di Camillo, Luisa Barzon, Gian Maria Fimia, Riccardo Manganelli, Eliana M. Coccia

**Affiliations:** 1 Department of Infectious Diseases, Istituto Superiore di Sanità, Rome, Italy; 2 Department of Molecular Medicine, University of Padova, Padua, Italy; 3 Department of Information Engineering, University of Padova, Padua, Italy; 4 Department of Surgery, Oncology and Gastroenterology, University of Padova, Padua, Italy; 5 National Institute for Infectious Diseases "L. Spallanzani”, Rome, Italy; 6 Department of Pathology, Institute for RNA Medicine, Beth Israel Deaconess Medical Center, Harvard Medical School, Boston, MA, United States of America; 7 Department of Biological and Environmental Science and Technology, University of Salento, Lecce, Italy; Portland VA Medical Center, Oregon Health and Science University, UNITED STATES

## Abstract

Autophagy is a primordial eukaryotic pathway, which provides the immune system with multiple mechanisms for the elimination of invading pathogens including *Mycobacterium tuberculosis* (Mtb). As a consequence, Mtb has evolved different strategies to hijack the autophagy process. Given the crucial role of human primary dendritic cells (DC) in host immunity control, we characterized Mtb-DC interplay by studying the contribution of cellular microRNAs (miRNAs) in the post-transcriptional regulation of autophagy related genes. From the expression profile of de-regulated miRNAs obtained in Mtb-infected human DC, we identified 7 miRNAs whose expression was previously found to be altered in specimens of TB patients. Among them, gene ontology analysis showed that miR-155, miR-155* and miR-146a target mRNAs with a significant enrichment in biological processes linked to autophagy. Interestingly, miR-155 was significantly stimulated by live and virulent Mtb and enriched in polysome-associated RNA fraction, where actively translated mRNAs reside. The putative pair interaction among the E2 conjugating enzyme involved in LC3-lipidation and autophagosome formation-ATG3-and miR-155 arose by target prediction analysis, was confirmed by both luciferase reporter assay and Atg3 immunoblotting analysis of miR-155-transfected DC, which showed also a consistent Atg3 protein and LC3 lipidated form reduction. Late in infection, when miR-155 expression peaked, both the level of Atg3 and the number of LC3 puncta per cell (autophagosomes) decreased dramatically. In accordance, miR-155 silencing rescued autophagosome number in Mtb infected DC and enhanced autolysosome fusion, thereby supporting a previously unidentified role of the miR-155 as inhibitor of ATG3 expression. Taken together, our findings suggest how Mtb can manipulate cellular miRNA expression to regulate Atg3 for its own survival, and highlight the importance to develop novel therapeutic strategies against tuberculosis that would boost autophagy.

## Author summary

*Mycobacterium tuberculosis* (Mtb) is one of the most successful pathogens in human history and remains the second leading cause of death from an infectious agent worldwide. The major reason of Mtb success relies on its ability to evade host immunity. Autophagy, a cellular mechanism involved in intracellular pathogen elimination, is one of the pathways hijacked by Mtb to elude the control of dendritic cells (DC), major cellular effectors of immune response. Recently, it has become clear that Mtb infection not only alters cellular gene expression, but also controls the level of small RNA molecules, namely microRNAs (miRNAs), which function as negative regulators of mRNA translation into protein. In the present study, we observed that the infection of human DC with Mtb leads to a strong induction of host miR-155, a critical regulator of host immune response. By mean of miR-155 induction, Mtb reduces Atg3 protein content, a crucial enzyme needed for the initial phase of the autophagic process. Interestingly, miR-155 silencing during Mtb infection restores Atg3 level and rescues autophagy. These findings contribute to better elucidate Mtb-triggered escape mechanisms and highlight the importance to develop host-directed therapies to combat tuberculosis based on autophagy boosting.

## Introduction

As estimated by the 2016 global WHO report, *Mycobacterium tuberculosis* (Mtb), the causative agent of tuberculosis (TB) in humans, infects approximately 10 million people each year, making TB the second leading cause of death from an infectious disease worldwide [1].

The success of Mtb principally relies on its ability to perfectly adapt to the host, by establishing latent infection and evading from the control driven by immune system. In this scenario, both innate and adaptive immune responses are required to control TB progression and pathogenesis [2].

In particular, the interaction of mycobacteria with antigen-presenting cells (APC) is a key feature in the pathogenesis of TB and the outcome of this interaction is pivotal in determining whether immunity or disease would ensue. Human and murine macrophages and dendritic cells (DC) were shown to be infected by mycobacteria and, in turn, to produce a specific immune response. As professional APC, DC display indeed an extraordinary capacity to stimulate naïve T cells and initiate primary immune responses [3, 4].

Once in contact with the pathogen, immature DC undergo maturation, modifying their phenotypic features and producing several pro-inflammatory and regulatory cytokines with the ability to tune the immune response by acting on different cellular populations [5]. These events result into a fine control exerted at transcriptional, post-transcriptional and post-translational level. In turn, host factors, utilized by mycobacterial molecules, play an important role in the regulation and progression of the infection [6].

In light of these observations, it is conceivable that Mtb infection might not only alter cellular gene expression but also control important regulators of mRNA translation, such as microRNAs (miRNAs). As a class of small non-coding RNAs, miRNAs are highly conserved within eukaryotic species and function as key regulators of gene expression by post-transcriptional targeting of mRNAs for translation arrest or for degradation [7]. These effects are mediated by imperfect binding of the miRNA-recognition elements within the 3' untranslated region (UTR) of the target mRNAs to the miRNA ‘seed’ region located between residues 2–8 at the 5' end [7].

In addition to the known role of miRNAs in the multi-level regulation of several cell processes and mammalian immune system [7], growing evidences show how pathogens profoundly perturb host miRNA expression. While the involvement of miRNAs in viral immune responses has been well investigated [8, 9], targeting of host miRNAs by bacterial pathogens is less characterized. Though, in recent years regulation of miRNA expression has been recognized as a molecular strategy exploited by bacteria to manipulate host cell pathways [10]. In particular, bacteria may redirect several host cell functions to sustain survival and/or replication. Most often, these effects require the delivery into the host cytoplasm of bacterial effector proteins through specific secretion systems [11]. Among closely related bacterial species, the mycobacterium genus is one of the most effective in inducing differential regulation of miRNAs [12, 13].

We set out to identify miRNAs perturbed by Mtb in infected human primary DC and understand their pathological relevance. We found that in human DC, Mtb induces miR-155 to negatively regulate ATG3, an E2-ubiquitin-like conjugating enzyme involved in autophagosome formation, thereby impairing autophagy.

## Results

### Identification of altered miRNAs in Mtb-infected DC

To investigate whether Mtb modulates the immune response by acting on the host DC miRNome, we perfomed a global analysis of miRNA expression profile in human primary DC infected with Mtb.

**Fig 1:**
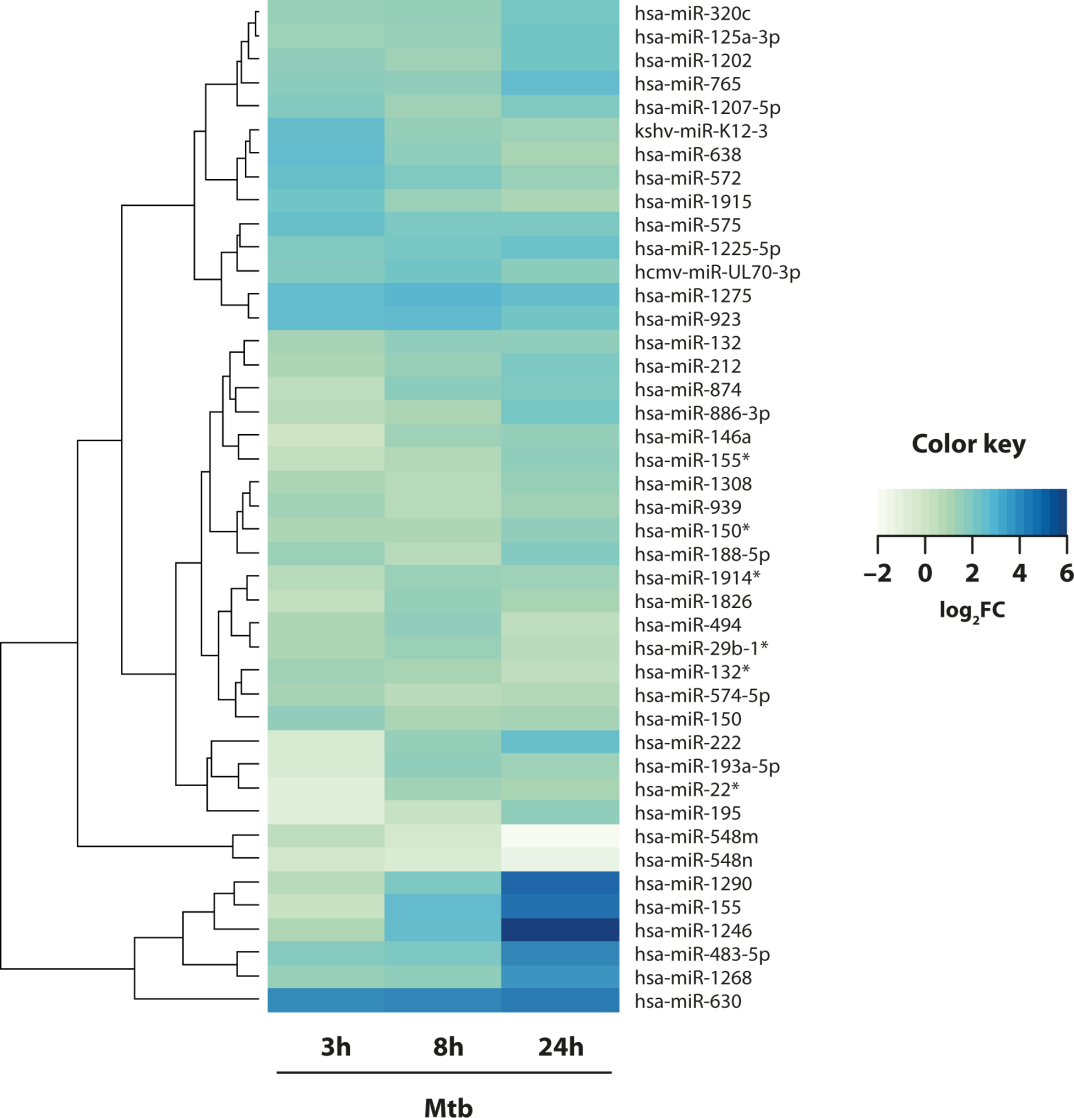
Identification of miRNAs altered in human DC during Mtb infection. DC were infected with Mtb for 3, 8 and 24 hours. Heatmap shows the significantly regulated miRNAs (FDR <0.05) during infection, in terms of log2 fold changes in the comparison Mtb-infected DC vs. untreated cells. Each row represents a single miRNA, averaged across different probes if significantly regulated (see Table 1), and each column a different time point, as indicated. The log2 fold change in the comparison Mtb-infected DC vs. CTRL are represented by a color code according to the legend. The dendrogram clusters together the significantly regulated miRNAs with most similar expression profiles.

RNA was prepared from DC infected in kinetic with live H37Rv Mtb for 3, 8 and 24 hours and microarray experiment performed. A hierarchical clustering was constructed by considering only miRNAs whose overall dynamic profile was significantly modulated in Mtb infected versus uninfected cells (Fig 1). The complete list of the 68 probes differentially expressed, corresponding to 43 different miRNAs is shown in Table 1. While only a few miRNAs were down-regulated as a consequence of Mtb challenge, the majority of them were found up-regulated in Mtb-infected DC (Fig 1). In particular, a high number of miRNAs showed a fold change (FC) greater than 2, starting from 3 hours post infection and in all the analyzed time-points, while only two miRNAs, namely miR-548m and miR-548n, were down-regulated with FC lower than -2 after 24 hours of Mtb infection (Fig 1).

**Table 1:** List of Mtb de-regulated miRNAs in DC.

probe ID	miRNA	p-value	FDR	FC (3 h)	FC (8 h)	FC (24 h)
A_25_P00010248	hsa-miR-630	5,55E-15	0	15,7	17,4	22,4
A_25_P00010249	hsa-miR-630	8,10E-15	3,12E-12	16,4	17,7	19,2
A_25_P00015143	hsa-miR-1246	3,62E-11	6,96E-09	1,8	6,1	64,7
A_25_P00012271	hsa-miR-155	1,54E-09	1,98E-07	1,2	8,2	29,2
A_25_P00014861	hsa-miR-483-5p	2,08E-09	2,00E-07	3,7	4,2	17,5
A_25_P00015210	hsa-miR-1275	2,47E-09	1,90E-07	6,1	7,2	5,9
A_25_P00013050	hsa-miR-923	4,38E-09	2,81E-07	6,8	7,7	4,3
A_25_P00015036	hsa-miR-320c	5,94E-09	3,27E-07	4,1	5,5	9,1
A_25_P00015195	hsa-miR-1268	6,67E-09	3,21E-07	2,8	2,9	24,5
A_25_P00015107	hsa-miR-1290	6,78E-09	2,90E-07	1,5	4,0	32,5
A_25_P00013051	hsa-miR-923	1,18E-08	4,54E-07	5,8	5,6	5,4
A_25_P00014896	hsa-miR-575	3,49E-08	1,22E-06	5,5	4,1	6,0
A_25_P00011342	hsa-miR-765	4,10E-08	1,31E-06	3,2	4,0	9,9
A_25_P00012270	hsa-miR-155	8,10E-08	2,40E-06	-1,1	4,5	23,9
A_25_P00014921	hsa-miR-1225-5p	1,21E-07	3,31E-06	4,1	4,3	5,6
A_25_P00014920	hsa-miR-1225-5p	1,80E-07	4,63E-06	3,8	4,4	5,3
A_25_P00010808	hsa-miR-575	5,37E-07	1,29E-05	5,7	4,1	2,9
A_25_P00015194	hsa-miR-1268	6,59E-07	1,49E-05	2,7	3,3	7,2
A_25_P00011724	hcmv-miR-UL70-3p	1,01E-06	2,17E-05	3,7	4,7	3,3
A_25_P00011096	hsa-miR-572	1,07E-06	2,16E-05	5,6	3,8	2,6
A_25_P00010402	hsa-miR-638	1,28E-06	2,46E-05	6,3	3,2	2,7
A_25_P00013690	kshv-miR-K12-3	1,86E-06	3,41E-05	6,5	2,9	2,6
A_25_P00012212	hsa-miR-125a-3p	2,28E-06	3,99E-05	2,9	3,4	4,7
A_25_P00015088	hsa-miR-1207-5p	3,49E-06	5,83E-05	4,0	2,6	4,1
A_25_P00012213	hsa-miR-125a-3p	4,41E-06	7,07E-05	2,8	2,5	5,5
A_25_P00013689	kshv-miR-K12-3	5,76E-06	8,87E-05	5,3	2,9	2,5
A_25_P00015075	hsa-miR-1202	7,04E-06	0,00010426	3,0	2,3	5,1
A_25_P00015076	hsa-miR-1202	1,04E-05	0,000148509	3,1	2,4	4,4
A_25_P00015087	hsa-miR-1207-5p	1,16E-05	0,00015901	3,5	2,3	4,0
A_25_P00010403	hsa-miR-638	1,16E-05	0,00015441	5,8	3,2	1,7
A_25_P00011341	hsa-miR-765	1,26E-05	0,000161445	3,4	2,3	3,9
A_25_P00010854	hsa-miR-212	1,84E-05	0,000227877	1,8	2,9	5,3
A_25_P00015303	hsa-miR-1915	2,49E-05	0,00029923	4,9	2,5	2,1
A_25_P00010589	hsa-miR-132	2,59E-05	0,000302001	2,0	3,2	3,9
A_25_P00012899	hsa-miR-886-3p	2,67E-05	0,000301895	1,6	3,0	5,1
A_25_P00015302	hsa-miR-1915	2,95E-05	0,000324597	4,9	2,6	1,9
A_25_P00013941	hsa-miR-125a-3p	8,50E-05	0,000908074	2,0	2,4	3,9
A_25_P00012126	hsa-miR-222	9,00E-05	0,00093585	-1,4	2,9	5,8
A_25_P00010588	hsa-miR-132	0,000113144	0,001145788	2,3	3,0	2,5
A_25_P00012919	hsa-miR-874	0,000123163	0,001215262	1,3	3,2	4,0
A_25_P00013453	hsa-miR-150*	0,000157478	0,001515006	1,7	2,5	3,8
A_25_P00010853	hsa-miR-212	0,000167906	0,001575933	2,0	2,5	3,2
A_25_P00012247	hsa-miR-188-5p	0,000187833	0,001720985	2,6	1,5	3,8
A_25_P00014847	hsa-miR-150	0,000350359	0,003135456	3,0	2,0	2,2
A_25_P00012898	hsa-miR-886-3p	0,000642922	0,005622907	1,3	1,4	5,7
A_25_P00015304	hsa-miR-1914*	0,000834574	0,007136873	1,6	2,7	2,4
A_25_P00014913	hsa-miR-886-3p	0,000880815	0,007368561	1,7	1,8	3,3
A_25_P00010079	hsa-miR-146a	0,000962151	0,007877726	-1,2	2,5	3,8
A_25_P00013087	hsa-miR-939	0,00131699	0,010558372	2,8	1,2	2,6
A_25_P00012254	hsa-miR-193a-5p	0,001583392	0,012435058	-1,5	3,2	2,4
A_25_P00015250	hsa-miR-1308	0,001598544	0,012302973	2,0	1,6	2,8
A_25_P00013255	hsa-miR-29b-1*	0,001775728	0,013398674	1,8	2,7	1,7
A_25_P00013450	hsa-miR-150*	0,002594343	0,019199043	2,2	1,5	2,3
A_25_P00015275	hsa-miR-1826	0,002610016	0,018950598	1,2	2,9	2,1
A_25_P00013086	hsa-miR-939	0,002618515	0,018660227	1,8	2,0	2,1
A_25_P00012505	hsa-miR-494	0,002908129	0,020347288	1,9	3,0	1,3
A_25_P00013400	hsa-miR-132*	0,003774944	0,025940486	2,0	2,0	1,7
A_25_P00013177	hsa-miR-22*	0,00387449	0,02615744	-2,1	2,3	2,1
A_25_P00010769	hsa-miR-195	0,004240248	0,028133179	-2,1	1,1	3,2
A_25_P00013472	hsa-miR-155*	0,004934236	0,032182776	1,2	1,7	3,1
A_25_P00013254	hsa-miR-29b-1*	0,005170278	0,033160285	1,9	2,4	1,3
A_25_P00013401	hsa-miR-132*	0,005214448	0,032895319	2,9	2,1	1,0
A_25_P00015184	hsa-miR-548m	0,006011955	0,037314673	1,3	-1,3	-4,0
A_25_P00015037	hsa-miR-320c	0,006540919	0,039953405	1,7	1,5	2,3
A_25_P00015182	hsa-miR-548n	0,00783043	0,047082686	-1,2	-1,7	-2,8
A_25_P00010078	hsa-miR-146a	0,007850726	0,046478497	1,1	2,4	2,2
A_25_P00012724	hsa-miR-574-5p	0,008522989	0,049693954	2,2	1,5	1,7

List of miRNA probes showing a statistically significant modulation of the overall dynamic profile in Mtb infected DC with respect to uninfected DC. FDR: false discovery rate; FC: fold change; 3 h: time 3 hours post-infection; 8 h: time 8 hours post-infection; 24 h: time 24 hours post-infection

*: miRNA star strand.

**Fig 2:**
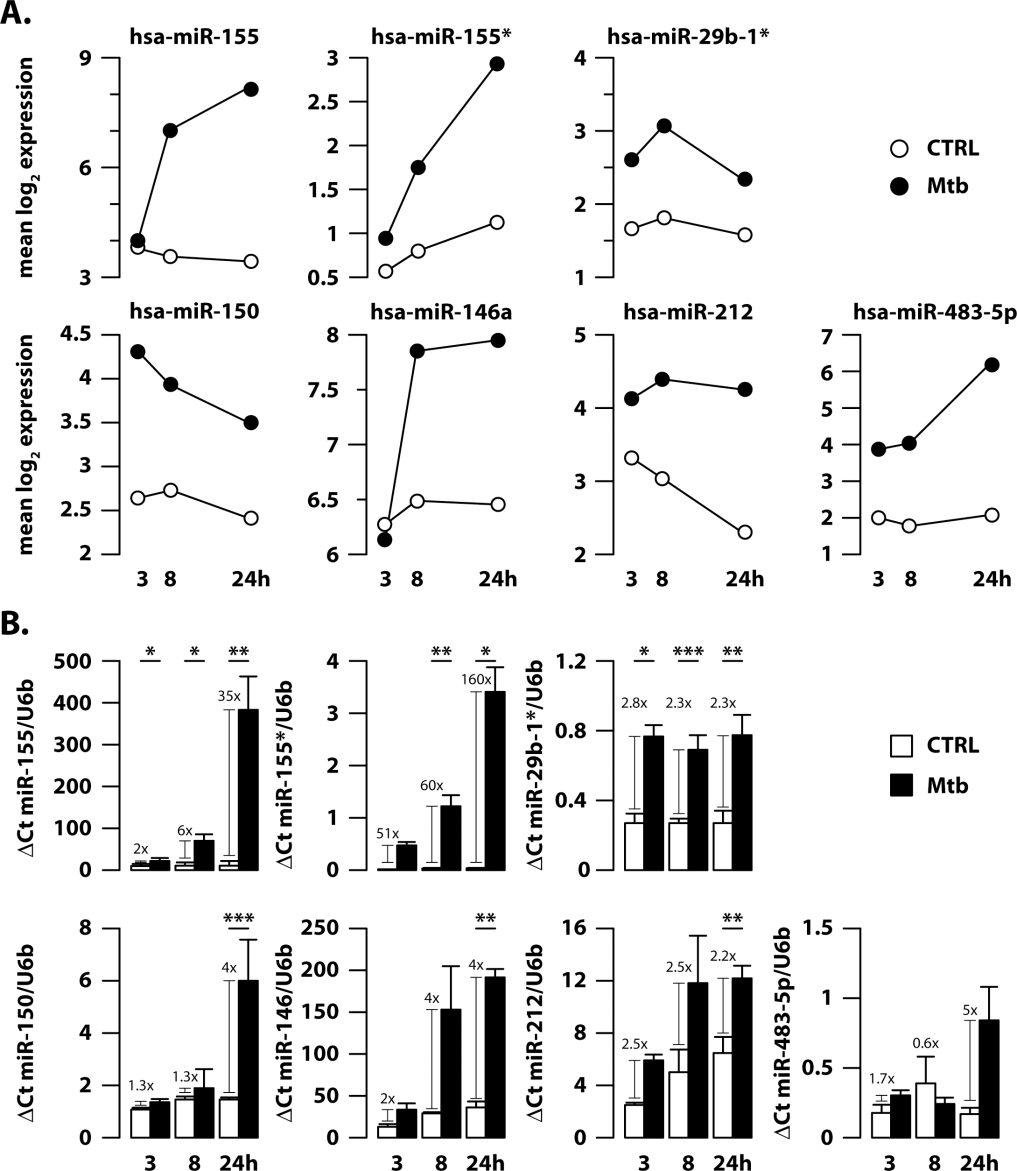
Validation of miRNAs altered in human DC during Mtb infection. DC were infected with Mtb for 3, 8 and 24 hours. (A) Time-series plots from array data of miR-155, miR-155*, miR-29b-1*, miR-150, miR-146a, miR-212 and miR-483-5p are shown. The selected miRNAs are all significantly up-regulated (FDR <0.05) during Mtb infection of human DC. When different probes corresponding to the same miRNA were found significant, the mean log_2_ expression profile across probes was plotted for the two conditions (CTRL and Mtb). (B) Relative expression of miR-155, miR-155*, miR-29b-1*, miR-150, miR-146a, miR-212 and miR-483-5p in human DC left untreated (CTRL) or infected for 3, 8 and 24 hours with wild type Mtb, as measured by q-PCR analysis. Data are derived from 3 healthy donors independently enrolled from those used for microarray and are normalized to the U6b level using the equation 2^-ΔCt^_._ The results shown were mean fold change per sample ± standard error of the mean at 3, 8 and 24 hours post-infection (*p = 0.005; **p = 0.02; ***p = 0.04).

To match our miRNA data with those observed in vivo during TB disease, the list of de-regulated miRNAs identified in infected DC was intersected with the miRNA profile found in patients affected by active TB [14–28]. Interestingly, among miRNAs de-regulated in TB patients and validated by real time PCR or similar techniques [14–28], seven miRNAs—namely miR-155, miR-155*, miR-29b-1*, miR-150, miR-146a, miR-212 and miR-483-5p –were found altered in Mtb-infected DC (Figs 1 and 2A). Furthermore, microarray data indicated that Mtb induced miR-29b-1*, miR-150, miR-212 and miR-483-5p at early time points (3 and 8 hours) (Figs 1 and 2A). MiR-150 and miR-212 induction was maintained over time, while 24 hour post-infection, miR-29b-1* decreased and miR-483-5p peaked (Fig 2A). Conversely, a different kinetic of expression was instead observed for miR-146a, miR-155 and its star filament, miR-155*, displaying an increasing level over time during Mtb infection (Fig 2A).

Microarray data were then validated by quantitative PCR analyses (q-PCR) in an independent cohort. According to microarray, miR-155, miR-155* and miR-146a were significantly induced by Mtb during human DC infection in a time dependent manner and, in particular, miR-155 and its star filament were among the top up-modulated miRNAs in our data set (Fig 2B).

On the contrary, although miR-29b-1*, miR-150, miR-212 and miR-483-5p induction was confirmed by q-PCR experiments, their kinetic of induction does not perfectly mirror that showed by microarray analysis (Figs 1 and 2). In particular, the levels of miR-212 and miR-483-5p induced by Mtb in infected DC and measured by q-PCR are weaker than those detected by microarray experiments while miR-29b-1* and miR-150 induction peaked at different time after Mtb infection when measured with the two techniques (Figs 1 and 2).

### Functional characteristics of putative targets of selected miRNAs

Since a single miRNA might regulate different mRNAs involved in distinct physiological processes, a functional gene ontology (GO) annotation study was conducted to identify those pathways and processes significantly represented within the list of targets of the seven selected miRNAs (for complete lists see S1 Table).

In particular, by taking advantage of the performed GO analysis, we noticed that, while miR-29b-1*, miR-150, miR-212 and miR-483-5p showed an enrichment in GO terms important for host-response to Mtb infection such as ubiquitin, proteasome degradation and endocytosis, only miR-155, miR-155* and miR-146a displayed a significant enrichment in biological processes linked to autophagy, a process deeply implicated in Mtb infection control [29, 30] (S2 Table).

### Specific induction of miR-155 expression by live Mtb

**Fig 3:**
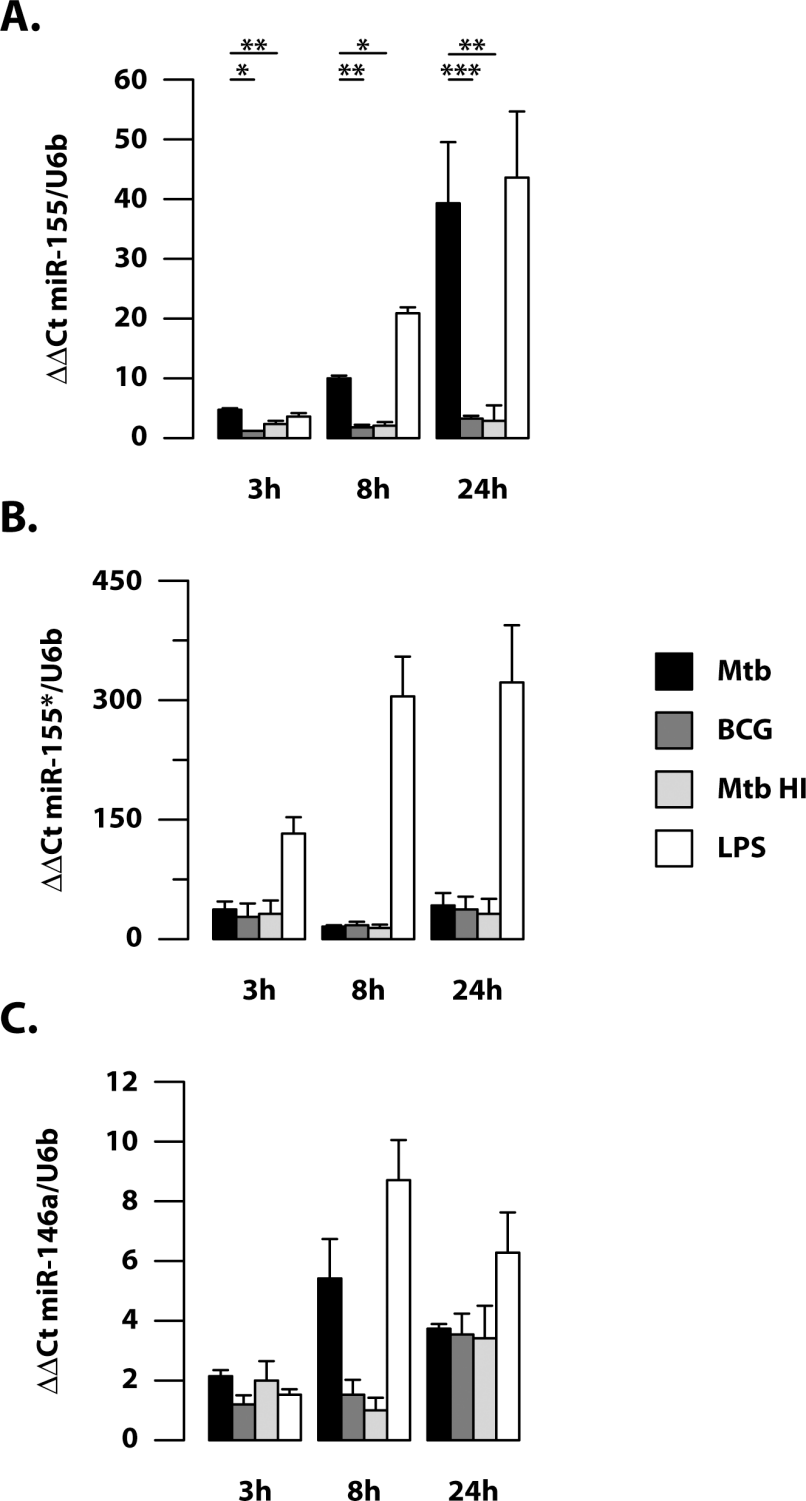
Modulation of miRNA expression in human DC infected with Mtb, BCG vaccine or treated with heat-inactivated Mtb and LPS. Relative expression of miR-155 (A), miR-155*(B) and miR-146a (C) in DC infected with Mtb or BCG vaccine or treated with heat-inactivated Mtb (Mtb HI) or LPS (1 μg/ml), as measured by q-PCR analysis. All quantification data are normalized to the U6b level using the equation 2^-ΔΔCt^. The results shown were mean fold change per sample ± standard error of the mean at 3, 8 and 24 hours post-infection of 3 healthy donors independently enrolled from those used in microarray study. (*p = 0.001; **p = 0.01; ***p = 0.02).

Having previously demonstrated that live and virulent Mtb is able to interfere with the autophagy pathway in human DC [29], we compared the expression of miR-155, miR-155* and miR-146a in DC stimulated with live or heat-inactivated Mtb (HI Mtb), the avirulent BCG vaccine, as well as LPS, as positive control (Fig 3).

While the expression of the three analyzed miRNAs was indistinctly promoted by LPS treatment, we interestingly found a different picture upon stimulation with the mycobacteria (Fig 3). Indeed, BCG and HI Mtb poorly induced miR-155 expression in treated DC as compared to live Mtb strain, suggesting that the modulation of miR-155 requires stimulation with virulent and live bacterium (Fig 3A). In contrast, miR-155* expression was similarly induced in response to both live and HI Mtb or BCG (Fig 3B). A different scenario was observed for miR-146a, induced in DC by all the analyzed stimuli at the different time points with no significant differences (Fig 3C).

Since the expression of miR-155 was preferentially stimulated by live and virulent Mtb strain, we focused on this miRNA and further investigated its function in the context of Mtb infection of human DC.

### Localization of Mtb-induced miR-155 into the polysome-associated RNA fraction

**Fig 4:**
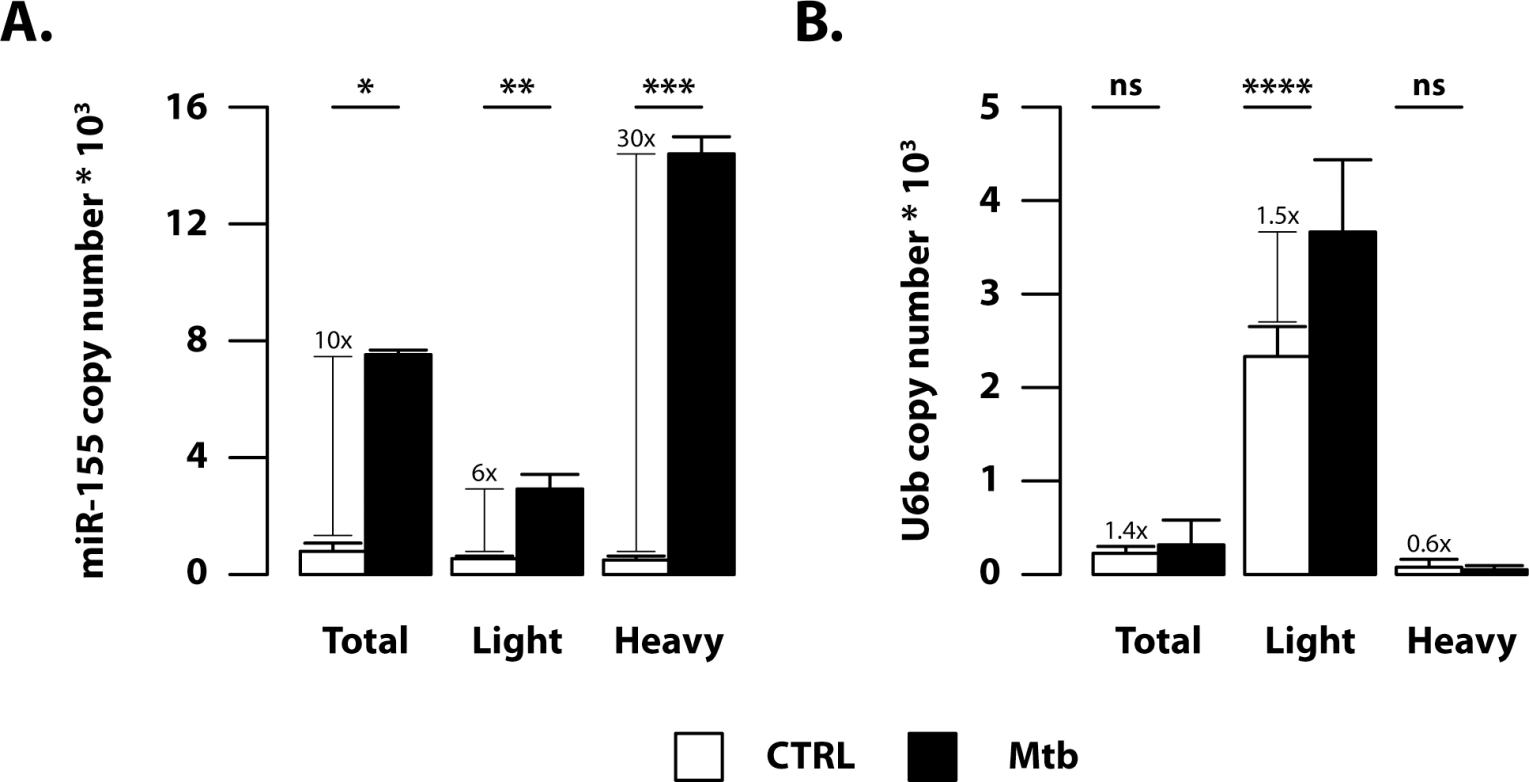
Mtb-induced miR-155 preferentially combines with polysome-associated mRNAs. Total RNA (Total), low occupancy (Light) and high occupancy (Heavy) polysome-associated mRNAs were extracted from untreated DC (CTRL) or DC infected for 16 hours with Mtb. (A) MiR-155 and (B) U6b copy number were determined by digital PCR. Data are represented as the mean copy number per sample ± standard error of the mean of three independent experiments.(*p = 0.00003; **p = 0.0008; ***p = 0.00009; ****p = 0.04).

Small RNAs, like miRNAs and RNA not associated with ribosome chains, principally reside in the light polysome fraction (constituted of monosome and single ribosome subunits), whereas actively translated mRNAs primarily occupy the heavy polysome fraction (rich in long ribosome chains). However, it has been recently shown that miRNAs can be co-purified with polysomes and their specific low (light fraction) or high (heavy fraction) polysome occupancy appears to reflect the strength of interaction with their target mRNAs [31]. Thus, we investigated whether the Mtb-driven miR-155 induction correlates with its localization in heavy polysome fraction by digital PCR approach (Fig 4). This allowed us to determine the absolute copy number of miR-155 in total as well as low and high occupancy polysome-associated RNA samples in the absence of a reliable housekeeping small RNA.

Interestingly, a 10-fold higher miR-155 copy number was found in total RNA samples from Mtb-infected DC and this increase was even higher (30-fold) in polysome-associated RNAs. Few miR-155 copies were instead associated to the light fraction of untreated and Mtb-infected DC (Fig 4A). As expected, the highest copy number of RNU6b - a commonly used housekeeping small RNA—was found in light fraction irrespective of Mtb infection (Fig 4B). Of note, after Mtb infection of human DC, the other two mycobacteria-induced miRNAs, namely miR-146a and miR-155*, mainly resided in light associated polysome fraction, although a low number of copies of those miRNAs was also present in heavy polysome associated RNA samples (S1 Fig). Taken together, these data indicate that upon Mtb infection the induced miR-155 is specifically associated with long ribosome chains where it may actively interact with its target mRNAs.

### Identification of ATG3 as a putative target for miR-155

**Fig 5:**
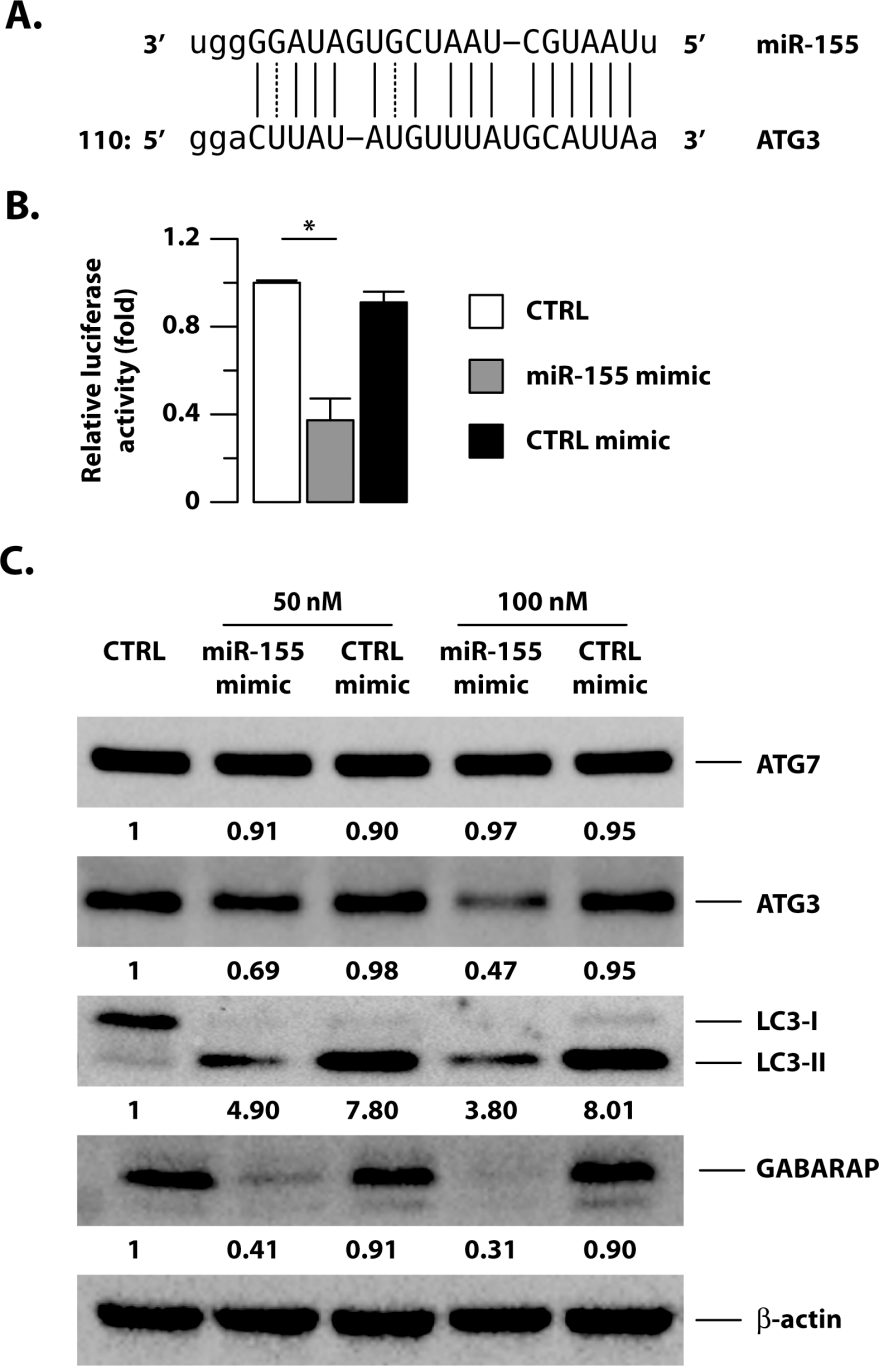
Identification and biological validation of ATG3 as miR-155 target in DC. (A) MiR-155 putative binding site in ATG3 3’-UTR region identified by multiple target prediction algorithms. (B) Luciferase assay on HEK293T cells showing specific targeting of 3′-UTR of ATG3 mRNA by the miR-155. Data are represented as the mean fold change per sample ± Standard Deviation of three independent experiments (*p = 0.018). (C) Atg7, Atg3, LC3 and GABARAP protein levels were determined by immunoblotting analysis on un-transfected DC (CTRL) or DC transfected for 24 hours with a miRNA mimic oligonucleotide for miR-155 and with a negative-control miRNA mimic (CTRL mimic). Band intensity quantification for Atg7, Atg3, LC3 and GABARAP is indicated at the bottom of each immunoblot. Actin levels were analyzed to verify the amount of loaded proteins. A representative experiment out of three experiments that yielded similar results is shown.

We performed an *in silico* target prediction analysis of miR-155 by employing and comparing five different algorithms: Targetscan (http://www.targetscan.org/), Pictar (http://pictar.mdc-berlin.de/), Diana-microT (http://diana.imis.athena-innovation.gr/DianaTools/index.php?r=microT_CDS/index), miRanda (http://www.microrna.org/microrna/home.do) and miRwalk (http://www.umm.uni-heidelberg.de/apps/zmf/mirwalk/index.html). In addition to several predicted and, in some case, validated targets of miR-155 [32–34], amongst the miR-155 targets, all 5 databases identified ATG3, an E2-ubiquitin-like-conjugating enzyme with an essential role in autophagosome formation [35, 36] (Fig 5A).

To validate the specificity of miR-155-ATG3 mRNA 3′-UTR pair interaction, a dual luciferase assay reporter vector containing the ATG3 3′-UTR sequence was transfected in HEK293 cells, together with a miR-155 mimic (hsa-miR-155-5p), a chemically synthesized, double-stranded RNA, which "mimics” the mature endogenous miR-155. A commercial random sequence miRNA mimic molecule was used as a negative control mimic. With this setting, a significant and selective reduction of luciferase activity was observed upon transfection with the miR-155 mimic, thus confirming a functional interaction between miR-155 and the ATG3 3’-UTR sequence (Fig 5B).

### MiR-155-driven reduction of ATG3 level and autophagosome number in Mtb infected DC

Next, we sought to correlate miR-155 expression with Atg3 protein levels, by studying the effects of the transfection of miR-155 mimic or negative control elicited in DC. Intriguingly, miR-155 mimic, but not the scrambled control, caused a dose-dependent reduction of Atg3 protein (about 30–50%), confirming that this enzyme is an authentic miR-155 target in human primary DC (Fig 5C).

As previously mentioned, Atg3 is an E2-ubiquitin-like-conjugating enzyme with a key role in the autophagy pathway, which, in a complex with the E1-ubiquitin-like-activating enzyme Atg7, transfers LC3 or GABARAP proteins to the phosphatidylethanolamine (PE) of the forming isolation membrane allowing their binding [37]. Accordingly, the low Atg3 protein abundance in miR-155 mimic-transfected DC correlated with the reduction of LC3-II and GABARAP (Fig 5C). Although the transfection procedure, requiring nutrient deprivation, *per se* promoted LC3 lipidation, a reduction of LC3-II was found only in response to miR-155 mimic transfection, while no effect was observed with the scrambled control. Interestingly, no variation in the abundance of Atg7 was observed, indicating that LC3-II reduction was strictly and specifically dependent on miR-155-mediated regulation of Atg3 expression (Fig 5C).

**Fig 6:**
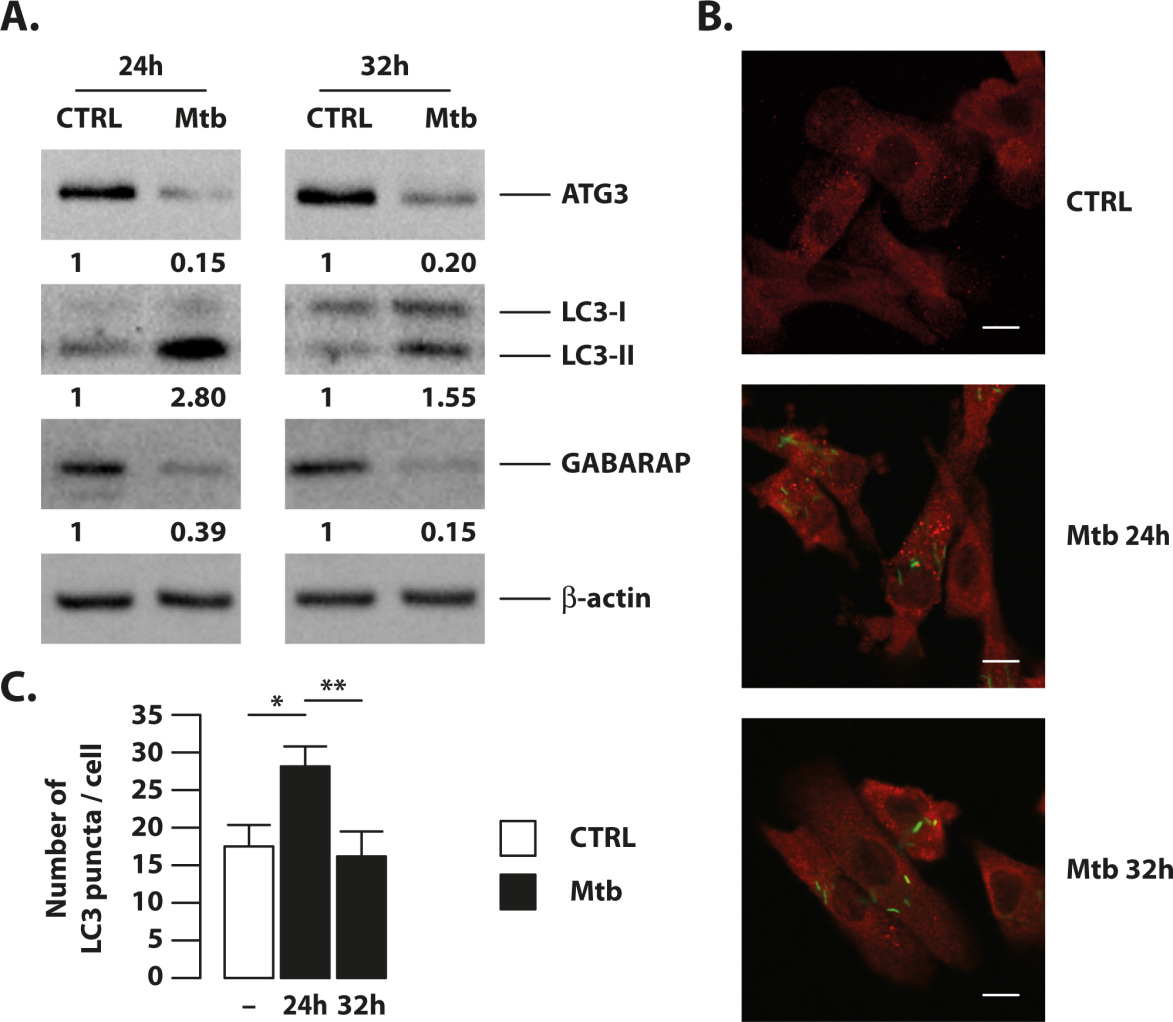
Mtb down-regulates Atg3 protein level and autophagosome number at later time point of infection. DC were infected with Mtb for 24 and 32 hours or left untreated (CTRL). (A) Atg3, LC3 and GABARAP levels were determined by Western blotting by using specific primary antibodies. Band intensity quantification is indicated at the bottom of each immunoblot. Actin levels were analyzed to verify the amount of loaded proteins. The results shown are representative of three independent experiments that yielded similar results. (B) Immunofluorescence analysis of the number of LC3 puncta per cell in Mtb-infected DC. DC were infected with Mtb for 24 and 32 hours or left untreated (CTRL), fixed and then stained with auramine to color Mtb bacilli (green) and with an anti-LC3 antibody (red). Representative images are shown in panel C. Scale bar 20 μm. (C) Graphic reporting a quantification of the number of LC3 puncta per cell performed as indicated in Material and Methods. The results represent the mean ± the standard error of the mean of three independent experiments (*p = 0.005; **p = 0.003).

Based on these findings, we next tested if Mtb-induced miR-155 would impair autophagosome formation by reducing Atg3 protein level and, in turn, affecting the capacity of DC to convert the autophagosome marker LC3 in its lipidated form. To this end, immunoblot analysis showed a significant decrease in the level of Atg3 24 hours after Mtb infection, which lasted up to 32 hours (Fig 6A), in tandem with increasing Mtb-driven miR-155 expression (S2 Fig). The reduction in Atg3 abundance driven by the infection correlated with an impairment of the lipidated form of LC3 (LC3-II) and GABARAP, particularly at 32 hours post-infection (Fig 6A). Furthermore, while the reduction in LC3 conversion was evident 32 hours post-infection, Mtb promoted LC3-II accumulation in 24 hour-infected DC, given its well-known ability to block autophagosome-lysosome fusion [29]. The variation in the abundance of LC3-II detected by immunoblotting was further confirmed by the significant decrease in the number of LC3 puncta per cell, as enumerated by confocal microscopy analysis in DC infected for 32 hours with Mtb as compared to those present in 24 hours-infected cells (Fig 6B and 6C).

**Fig 7:**
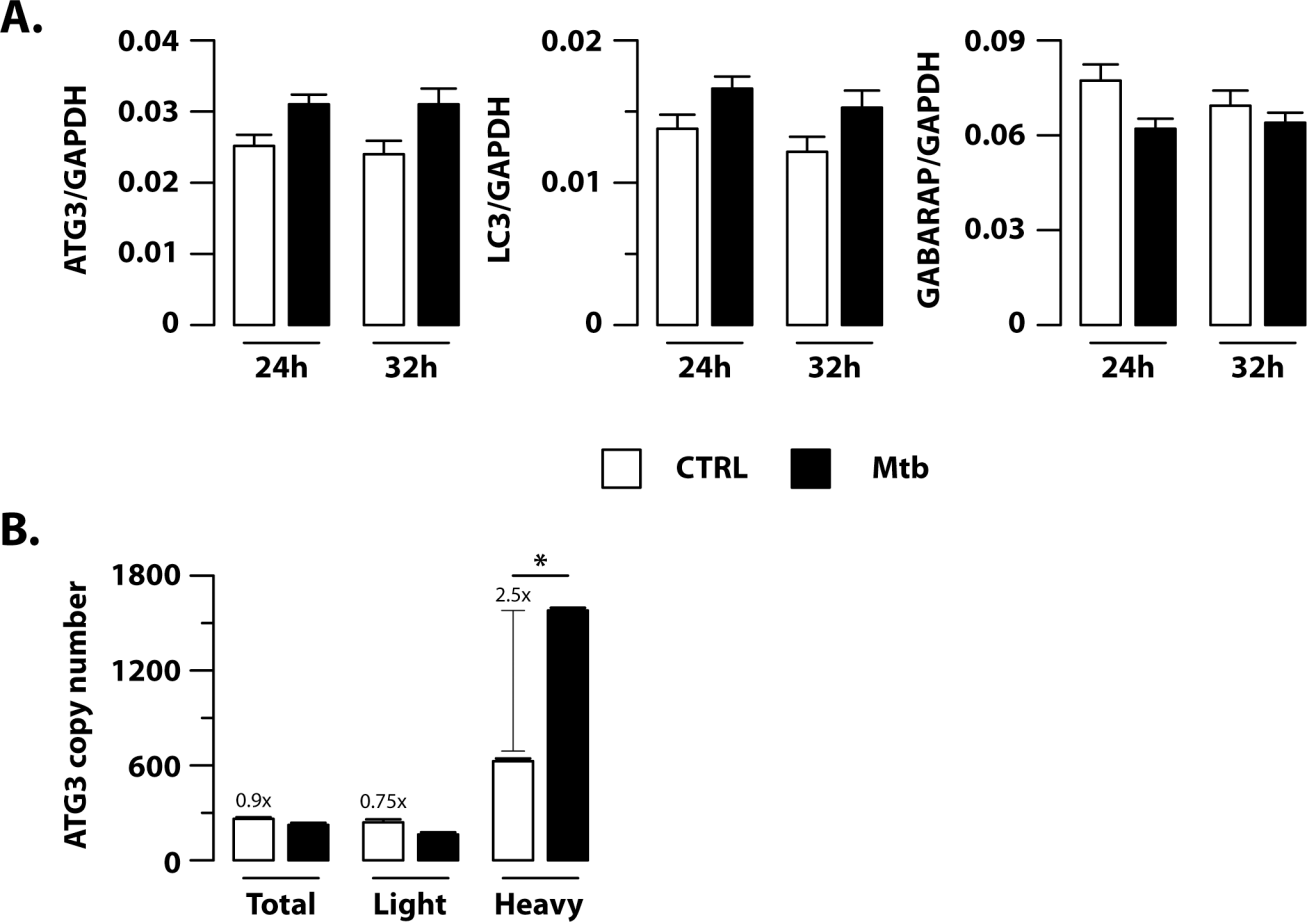
Mtb infection favors ATG3 transcript association with polysome chains in DC. (A) Relative expression of ATG3, LC3 and GABARAP in human DC infected with Mtb or left untreated (CTRL), as measured by q-PCR analysis on extracted total RNA samples. All quantification data are normalized to the GAPDH level using the equation 2^-ΔCt^. The results are the mean fold change per sample ± standard error of the mean at 24 and 32 hours post-infection of three experiments. (B) Total RNA (Total), low occupancy (Light) and high occupancy (Heavy) polysome-associated mRNAs were extracted from untreated DC (CTRL) or DC infected for 16 hours with Mtb. ATG3 copy number was determined by digital PCR. Data are represented as the mean copy number per sample ± standard error of the mean of three independent experiments (*p = 0.004).

Since expression of ATG3, LC3 and GABARAP mRNAs was unchanged upon Mtb challenge (Fig 7A), it is likely that the miR-155 mediated reduction of Atg3 impacts at a post-transcriptional level on LC3 and GABARAP processing and, in turn, on autophagosome formation. In line with q-PCR data, no variation in ATG3 mRNA copy number was detected by using digital PCR on total RNA samples from Mtb-infected DC as compared to the untreated counterpart (CTRL) (Fig 7B). Conversely, association of ATG3 mRNA to high occupancy polysome chains was significantly induced by Mtb infection as evident from the 2.5 fold induction of ATG3 expression measured in polysome-associated RNA fraction (Fig 7B). Interestingly, our analysis highlighted that Mtb infection favors the association of both miR-155 and ATG3 mRNA on polysome chains (Figs 4A and 7B), thus supporting the miR-155-mediated regulation of ATG3 protein level in DC during Mtb infection.

### Rescued autophagosome formation and autolysosome fusion in Mtb-infected DC by miR-155 silencing

**Fig 8:**
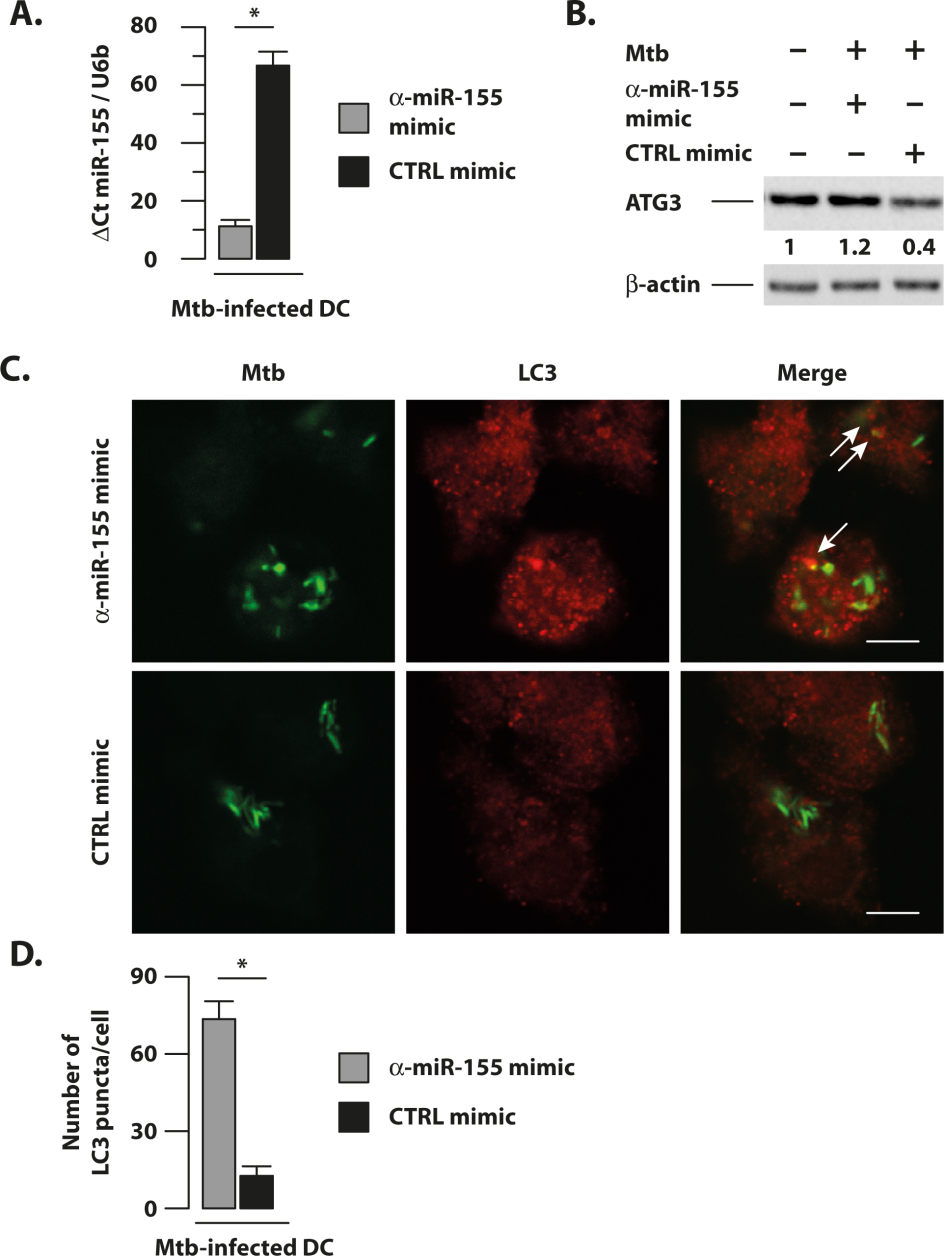
Silencing of Mtb-induced miR-155 expression restores DC ability to form autophagosome. DC were left untreated (CTRL) or infected for 8 hours with Mtb at MOI of 1 bacterium/cell and then transfected with 100 nM of an antagomir oligonucleotides for miR-155 (α-miR155 mimic) or with a negative control mimic (CTRL mimic) for 24 hours. (A) Silencing of miR-155 expression was verified by qPCR analysis on total RNA samples. The results represent the mean ± the standard error of the mean of three independent experiments (*p = 0.03). (B) Atg3 protein levels were determined by immunoblotting analysis. Band intensity quantification for Atg3 is indicated at the bottom of each immunoblot. Actin levels were analyzed to verify the amount of loaded proteins. The results shown are from one of three experiments that yielded similar results. (C) Immunofluorescence analysis of the number of LC3 puncta per cell in DC infected with Mtb for 8 hours and then transfected with an antagomir oligonucleotides for miR-155 (α -miR155 mimic) or with a negative control mimic (CTRL mimic). Thirty-two hours after infection cells were fixed and then stained with auramine to color Mtb bacilli (green) and with an anti-LC3 antibody (red). Co-localization of LC3 puncta with Mtb bacilli is indicated by white arrows. A representative image for each condition is shown in panel C. Scale bar 15 μm. (D) Graphics reporting a quantification of the number of LC3 puncta per cell performed as indicated in Material and Methods. The results represent the mean ± standard error of the mean of three independent experiments (*p = 0.04).

**Fig 9:**
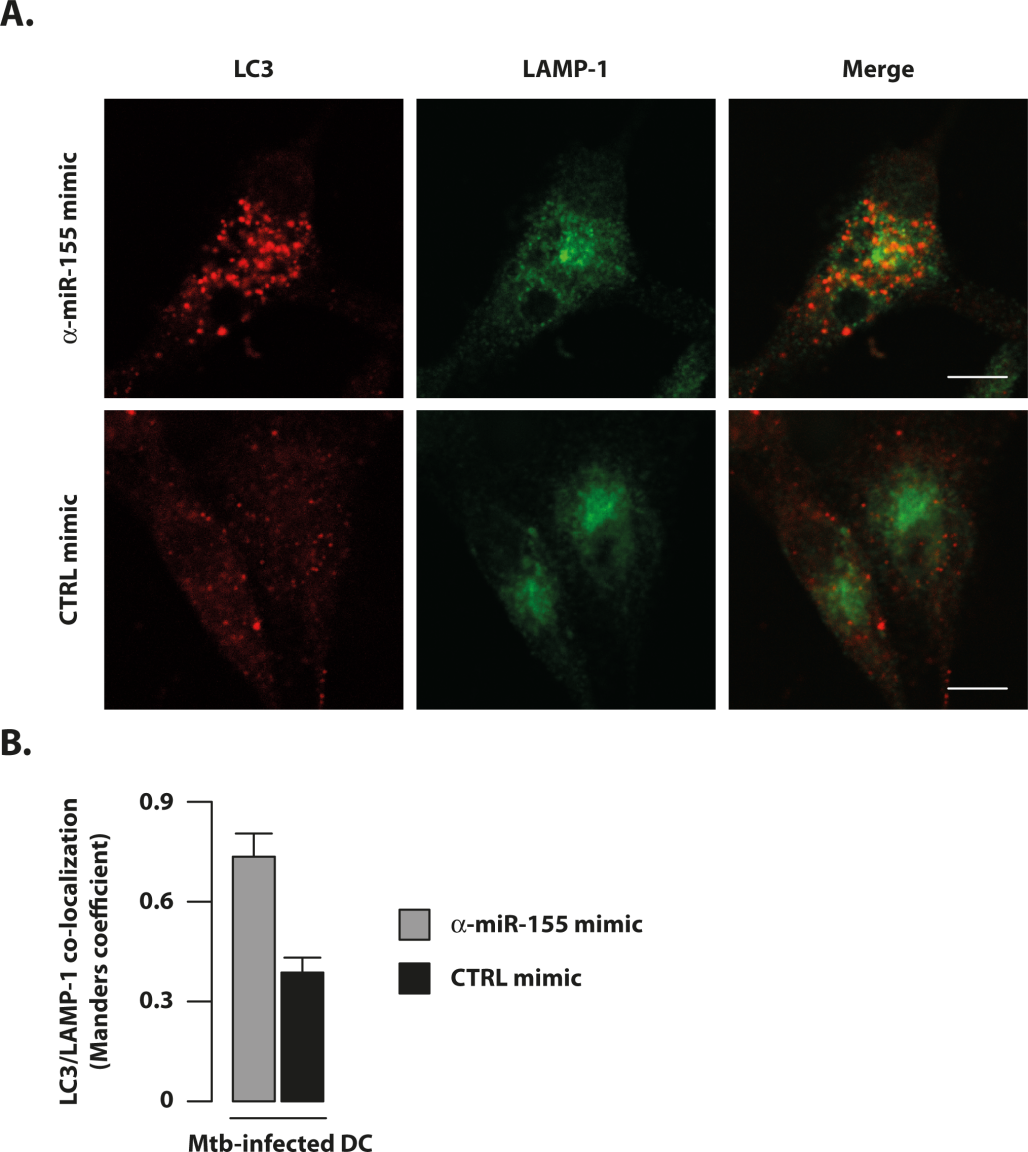
Silencing of miR-155 during Mtb infection recovers DC autophagic flux. DC were infected for 8 hours with Mtb at MOI of 1 bacterium/cell and then transfected with 100 nM of an antagomir oligonucleotides for miR-155 (α-miR155 mimic) or with a negative control mimic (CTRL mimic) for 24 hours. (A) Immunofluorescence analysis of LC3 and LAMP1 in DC infected with Mtb for 8 hours and then transfected with an antagomir oligonucleotides for miR-155 (α -miR155 mimic) or with a negative control mimic (CTRL mimic). Thirty-two hours after infection cells were fixed and then stained with anti-LC3 (red) and anti-LAMP1 (green) antibodies. A representative image for each condition is shown in panel A. Scale bar 15 μm. (B) Graphics reporting a quantification of the experiment described in A and performed as indicated in Material and Methods. The results represent the mean ± standard error of the mean of two independent experiments.

To test whether silencing of Mtb-induced miR-155 impacted on autophagy, Mtb-infected DC were transfected with a specific inhibitor of miR-155 (α-miR-155 mimic) or with a non-specific miRNA inhibitor (CTRL mimic) (Fig 8). Silencing efficiency was assessed by analyzing the level of miR-155 expression in infected cells. An important (≈75%) reduction was observed only in the presence of the specific inhibitor (α-miR-155 mimic) (Fig 8A). As expected, by antagonizing Mtb-induced miR-155 expression in infected DC, the ATG3 protein content was restored at a level similar to that found in untreated and un-transfected control cells (Fig 8B). Moreover, following the specific inhibition of miR-155, DC recovered their capacity to form autophagosomes in response to Mtb infection, as demonstrated by the significant increase in the number of LC3 puncta per cell (Fig 8C and 8D). Notably, the selective silencing of miR-155 in Mtb-infected DC consistently stimulated the fusion of rescued autophagosomes with lysosomes as demonstrated by the increased co-localization of the LC3 signal with the lysosomal marker, LAMP-1, thus, allowing the recovery of the Mtb-blocked autophagic flux (Fig 9).

## Discussion

During their coevolution with the host, successful pathogens, like Mtb, have evolved a myriad of strategies to evade and, in some cases, subvert innate and adaptive immune responses [6]. These include targeting of autophagy, a cellular mechanism usually devoted to recycle damaged organelles or macromolecules, which also plays a crucial role in counteracting continuous microbial insults [37]. More specifically, the importance of the autophagic process in host immune response against Mtb has been demonstrated by the fact that virulent mycobacteria may impair phagosome maturation by altering the acidic, hydrolytic environment of the intracellular compartment needed to kill the bacteria, thus promoting their own survival [30]. Moreover, the impairment of autophagy machinery compromises antigen-processing capacity [38, 39] thus, affecting specific host-immune response. Lastly, in Mtb-infected human DC, the block of autophagosome maturation could be overcame by the autophagy inducer, rapamycin, which simultaneously boost a protective Th1 response [29].

In spite of the fact that autophagy has been a fast-growing research area in recent years, not much was known about immune-evasion mechanisms exploited during both viral and bacterial infections and acting on the E2-like conjugating enzyme Atg3, critical for LC3 lipidation during autophagosome formation [37]. Here, we show for the first time the capacity of Mtb to manipulate autophagy, by impairing autophagosome formation, through the host miR-155-mediated targeting of ATG3 in infected human DC.

The mechanism described in the present study is also in support of the involvement of the Mtb-triggered de-regulation of host miRNA profile, which, in turn, interferes at post-transcriptional level with the fine-tuned DC gene expression program, crucial for the establishment of an appropriate immunological outcome [13].

In addition to miR-155, we identified several others miRNAs induced in Mtb infected DC, as miR-155*, miR-29b-1*, miR-150, miR-146a, miR-212 and miR-483-5p, whose expression was also found altered in patients affected by pulmonary TB [14–28]. A mechanistic explanation concerning the function of some of these miRNAs along mycobacterial infection has been already proposed in previous in vitro studies [40–45]. In particular, Mtb stimulates the expression of miR-29b-1* to target IFN-γ in both NK and CD4+ T cells [40]. A negative regulation of the TLR2 signaling was instead demonstrated for miR-150 although in the context of BCG infection [41]. A dual function was shown for miR-146a, which acts on PTGS2, thus increasing macrophages ability to kill BCG, and reduces TNF-α release by targeting both IRAK1 and TRAF6 [42, 43]. Finally, several roles were proposed for miR-155 in the context of mycobacterial infection in both innate and adaptive immune cells spanning from maintenance of cell survival to interference with the autophagy pathway [44–46].

Given the critical role played by autophagy in Mtb infection control [47], we performed a GO analysis to get insight into the potential functions of Mtb de-regulated miRNAs with a particular focus on this cellular process. The analysis of putative miRNA target lists revealed a significant enrichment in autophagy-linked biological processes for three of the selected miRNAs, namely miR-155, miR-155* and miR-146a.

Among these miRNAs, we focused our attention on miR-155 because its expression was specifically induced by live Mtb, and not by HI Mtb or by the vaccine strain BCG. This feature is particularly interesting in light of our previous data showing that DC differentially respond to live, dead or attenuated Mtb by promoting the expression of a specific cytokine milieu and by modulating the autophagic process [5, 29, 48].

Although the potential correlation between miR-155 and mycobacterial infection was already proposed [13, 17, 32, 44], the presence of miR-155 into the polysome-associated RNA fraction is a novel finding supporting the idea that in this subcellular localization miR-155 can engage actively translated mRNA transcripts to specifically control gene expression in Mtb-infected DC.

Interestingly, data obtained so far in human DC are in support of the interaction between miR-155 and ATG3 indicated by our *in silico* target prediction analysis. Indeed, we found a decrease in Atg3 protein level at late time points of Mtb infection together with the impairment of LC3 puncta number per cell. Notably, LPS treatment does not alter LC3-II level in human DC in spite of its ability to strongly induce miR-155 expression (S3 Fig), thereby uncovering a previously unidentified Mtb-driven effect of miR-155 on ATG3 expression.

MiR-155-dependent control of ATG3 expression was confirmed by luciferase assay and by transfection experiments conducted in human primary DC with a specific miR-155 mimic and showing that miR-155 down-regulates Atg3. This impairment translates into a reduced ability of the complex Atg7/Atg3 to convert LC3-I in LC3-II and in a diminished capacity to process the LC3 homologue, GABARAP. Indeed, Atg7, which was not altered as a consequence of miR-155 mimic transfection, activates and transfers LC3-I to the E2-ubiquitin-like-conjugating enzyme, Atg3, essential for the final conjugation of LC3 to the PE group [49]. These events allow the assembling of the membrane-bound form, LC3-II, to the newborn autophagosome structures.

Notably, by using an anti-miR-155 mimic, we were able to restore Atg3 protein and its capacity to form autophagosomes during Mtb infection of human DC. Importantly, the silencing of miR-155 rescued autophagic flux commonly altered in DC as a consequence of Mtb infection. These data strongly support the role of miR-155 in controlling the autophagic process in human DC and suggest the miR-155/ATG3 axis as a critical component in the management of autophagosome formation as well as of the autophagic flux during Mtb infection.

In addition to the mechanism here demonstrated for Atg3 protein, it has been reported that another autophagy protein involved in autophagosome formation, namely Atg5, has an autophagy-independent protective function in neutrophil-mediated immunopathology during Mtb infection, however, its role seems to be dispensable in myeloid cells [50].

It was recently demonstrated that the miR-155-mediated regulation of the survival pathway in innate and adaptive immune cells leads to opposite impacts with regard to Mtb containment [44]. Accordingly, depending on cell type and stimuli, miR-155 might act as inducer as well as repressor of the autophagic process [45, 51–53]. Indeed, a pro-autophagic function for miR-155 was shown by its capacity to target multiple players within the mTOR cascade as Rheb, although in response to hypoxia or to infection with avirulent Mtb or BCG vaccine [45, 51]. Conversely, Holla and colleagues demonstrated that miR-155 limits IFN-γ-induced autophagy in murine macrophages infected with BCG or Mtb [52]. This is consistent with a negative role for miR-155 in human DC infected with Mtb, as proposed in the present study. In line with this view, it has been suggested that inhibition of this miRNA hinders Mtb survival [53]. This further underlines that Mtb might induce miR-155 to its own end. Moreover, it was also demonstrated that in addition to miR-155, Mtb engages other miRNAs, such as miR-33 and its star filament miR-33*, to limit the autophagic pathway, whose up-regulation in infected macrophages leads to repression of multiple autophagy effector molecules [54]. The notion that Mtb might use cellular miRNAs to interfere with autophagy process has been also confirmed by other recent studies showing that Mtb-mediated alteration of miR-125a, miR-17 as well as miR-144* expression levels undermines the anti-microbial activity of autophagy in infected macrophages or monocytes [55–57].

**Fig 10:**
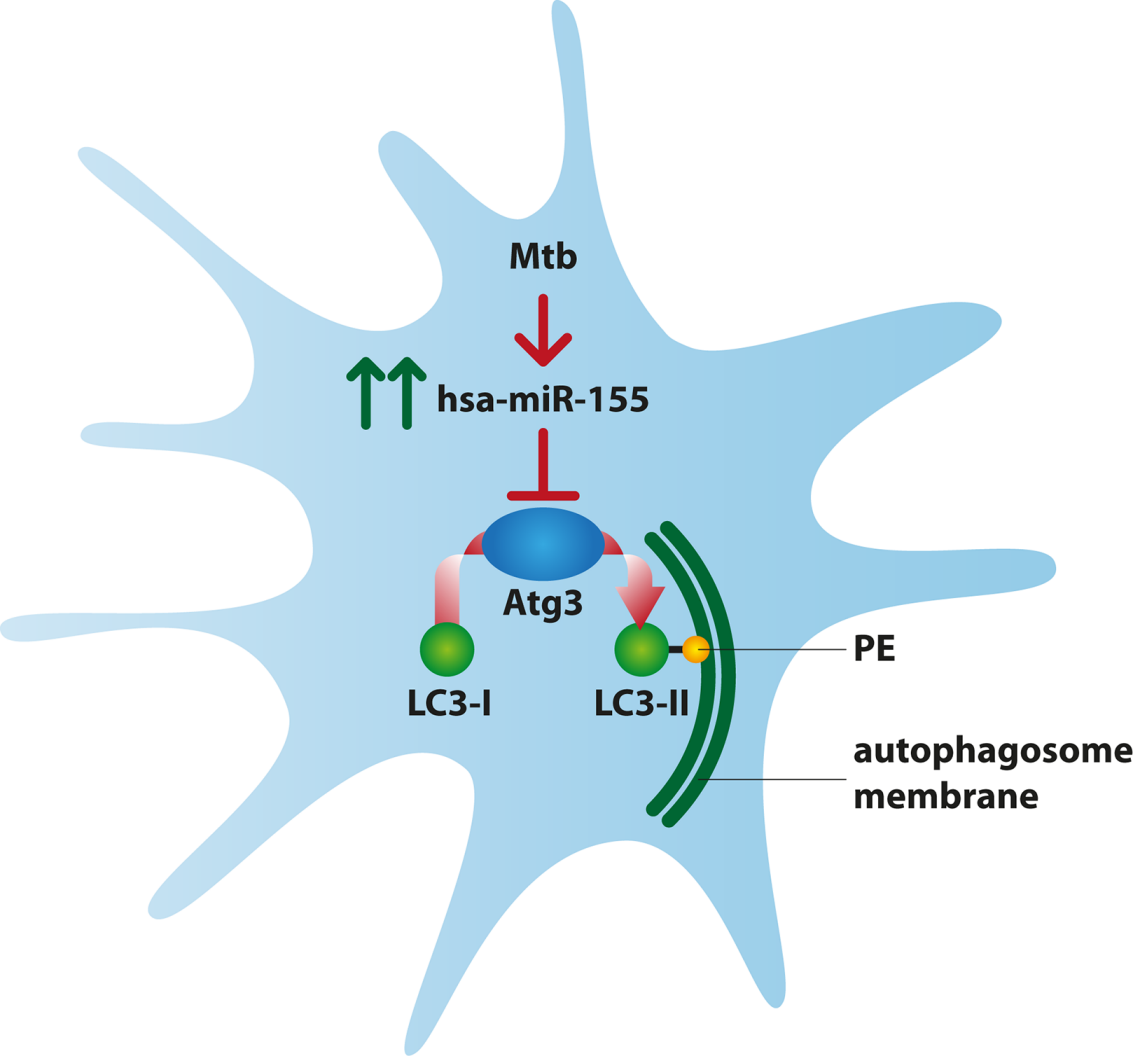
Model for Mtb-triggered inhibition of Atg3 activity. Mtb infection of human primary DC causes the strong induction of hsa-miR-155 expression. The de-regulated miR-155 interacts with the 3’UTR region of the ATG3 mRNA, leading to a reduced protein level. As a consequence, an impaired LC3 conversion into its lipidated form (LC3-II) through the addiction of the phosphatidylethanolamine (PE) was observed in Mtb-infected DC.

Based on these findings, it is clear that Mtb can manipulate the expression of cellular miRNAs in order to enhance its survival inside the host and, our study, highlights a novel Mtb-triggered escape mechanism involving cellular miR-155 by which Mtb antagonizes the autophagic process via a previously unidentified ATG3 reduced expression (Fig 10).

Taken together our data suggest that reconstitution of normal autophagy by antisense miR-155 molecules could represent an innovative approach for exploiting novel host-directed therapeutic strategy to combat TB.

## Methods

### Antibodies (Abs) and other reagents

Monoclonal Abs specific for CD1a, CD14, CD86 (BD Bioscience, San Jose, CA, USA) and Fixable Viability Dye (FvDye, eBioscience, San Diego, CA, USA) were used as direct conjugates to fluorescein isothiocyanate (FITC), phycoerythrin (PE) or eFluor780 as needed.

For immunoblotting analysis, goat anti-Atg7 (Santa Cruz Biotechnologies, Santa Cruz, CA, USA), rabbit anti-Atg3 (Cell Signaling, Danvers, MA, USA), rabbit anti-GABARAP (Santa Cruz Biotechnologies), rabbit anti-LC3 (Cell Signaling), mouse anti-actin (Sigma-Aldrich, St. Louis, MO, USA), and horseradish peroxidase-conjugate secondary anti-goat, anti-mouse and anti-rabbit Abs (Santa Cruz Biotechnology) were used.

Where indicated, LPS (Sigma-Aldrich) was used at 1 μg/ml.

### Ethics statement

Istituto Superiore di Sanità Review Board approved the present research project (CE/13/387). No informed consent was given since the data were analyzed anonymously. Peripheral blood mononuclear cells were isolated from freshly collected buffy coats obtained from healthy voluntary blood donors (Blood Bank of University "La Sapienza", Rome, Italy).

### DC preparation and cell culture

DC were prepared as previously described [5]. Briefly, DC were generated by culturing CD14^+^ monocytes with 50 ng/ml GM-CSF and 1000 U/ml IL-4 (R&D Systems, Minneapolis, MN, USA) for 5 days at 0.5x10^6^ cells/ml in RPMI 1640 (Lonza, Basel, Switzerland), supplemented with 2 mM L-glutamine and 15% Fetal Bovine Serum (FBS) (BioWhittaker, #DE14-801F). At day 5 the cells were tested for their differentiation status by evaluating CD1a expression (>90% CD1a^+^) and lack of CD14 (>95% CD14^-^). Before infection, the medium was replaced with RPMI without antibiotics and supplemented with 2 mM L-glutamine and 15% FBS. Cytokine deprivation did not affect DC survival rate, which was >90%.

For gene reporter assay, HEK293T cells (5x10^5^ for each condition; a generous gift of Dr. Kate A. Fitzgerald—University of Massachusetts Medical School, Worcester, MA) were re-suspended in 100 μl DMEM (Lonza) plus 10% FBS (BioWhittaker without antibiotics and plated in a 96-well plate.

### Bacterial strain description and preparation

Mtb H37Rv (ATCC 27294; American Type Culture Collection) was grown as previously described [5]. Logarithmically growing cultures were centrifuged at 800 rpm for 10 min to eliminate clumped mycobacteria and then washed three times in RPMI 1640. Mycobacteria were re-suspended in RPMI 1640 containing 10% FBS and then stored at -80°C. Bacterial frozen vials were thawed and bacterial viability was determined by counting the number of colony forming units. All bacterium preparations were tested for endotoxin contamination (<1 Endotoxin Unit/ml) by the Limulus lysate assay (Lonza). DC cultures were then infected with a multiplicity of infection (MOI) of 1 bacterium/cell as previously described [5]. Where indicated, Mtb was heat-killed at 80°C for 1 hour.

### Total RNA extraction, quantification ad quality assessment

Total RNA was isolated from 1x10^6^ infected or not infected DC using mirVana isolation kit (Ambion, Thermo Fisher Scientific, Waltham, MA, USA) and following the manufacturer’s recommendations. An additional phenol/chloroform extraction was performed to inactivate residual mycobacterial particles. RNA was quantified using a Nanodrop spectrophotometer (Nanodrop2000, Thermo Fisher Scientific) and quality assessed with an established cut-off of 1.8 for both the 260/280 and 260/270 absorbance ratios. RNA integrity was instead inspected by bioanalyzer analysis (Agilent Technologies, Santa Clara, CA, USA) considering a cut-off of 1.8 for 28S/18S ratio.

### miRNA microarray analysis

Microarray experiments were carried out on the Human miRNA Microarray platform (V3) (Agilent Technologies). 100 ng of total RNA of experimental sample (infected DC) and of a reference sample (uninfected DC) was labeled with cyanine Cy3 (miRNA Complete Labeling and Hybridization Kit, Agilent Technologies). The labeled experimental and reference samples were denatured and then hybridized on microarray slides containing microRNA capture probes targeting all human and viral miRNAs listed in the Sanger miRBase database, release 12.0. Each slide was placed in a hybridization chamber (Agilent # G2534A) and hybridized in a hybridization oven (Agilent # G2545A) for 20 hours at 55°C. Low- and high-stringency washes were carried out and the microarrays were dried following the Agilent washing protocol. A G2565C microarray scanner (Agilent Technologies) was used to acquire images and Feature Extraction software version 10.1 (Agilent Technologies) was used to quantify hybridization signals. Absent and marginal signals, flagged automatically by the software, were removed from the analysis. Probe intensities were log2-transformed, after adding an offset to accommodate zeros according to the formula [x′ = log2(x+1)], where x′ is the adjusted signal and x the ‘total probe signal’ calculated by Feature Extraction software. Normalization was executed according to the quantile normalization method [58]. Quality assessment was based on both the Feature Extraction Quality Control reports and the pre- and post-normalization MA plots, which allow a pairwise comparison of log-intensities of each array to a reference median array and also the identification of intensity dependent biases. Samples showing a poor reproducibility of the replicate probes or/and evident trends or saturation effects in the corresponding MA plots were excluded from the analysis.

To test for miRNA differentially expressed between CTRL and Mtb-infected DC we applied the “Bounded Area” method [59, 60] to identify the miRNA probesets whose overall dynamic profile is significantly modulated in Mtb.

Briefly, the area bounded by the miRNA profile with respect to its baseline value was evaluated, and the miRNA was considered differentially expressed if this area exceeded a threshold θ. θ is the confidence threshold determined in correspondence to a significance level α, based on the null hypothesis distribution. This latter was derived by repeatedly sampling data from an error distribution, empirically obtained from the available biological replicates as explained in [61].

As already shown, the method is quite robust to random oscillation since the entire expression profile is considered, thus diminishing both false positive and false negative rates with respect to standard tests when few replicates are available. Moreover, both the precision and the recall of the test are enhanced by the fact that the entire expression profile is tested, rather than single time points. In order to account for multiple testing, the significance level α was corrected for multiple testing so to control the global False Discovery Rate at 5%.

MiRNA microarray data have been deposited in Array Express public repository (https://www.ebi.ac.uk/arrayexpress/, accession number: E-MTAB-6083) according to the MIAMEstandards. Hierarchical clustering was performed using Euclidean distance and average linkage method. Bioinformatics analysis was implemented in the R statistical environment.

### Isolation of polysome-associated RNA

For RNA fraction isolation, 10x10^6^ cells untreated DC (CTRL) and DC infected for 16 hours with Mtb (MOI 1) were used. Polysome-associated RNA were prepared as previously described [62].

Each gradient was fractionated in BSL3 facility by hand collection from the top of the gradient into 2 samples, light phase (low polysome occupancy) and heavy phase (high polysome occupancy). The hand collection method was previously tested to parallel with fraction collection using a fractionator coupled with an UV optical reader (A 254nm). The obtained fractions were immediately mixed with an equal volume of Trizol (Invitrogen, Thermo Fisher Scientific) for later RNA isolation following Trizol manufacturing instructions.

### Quantitative real time PCR and digital PCR

Total RNA was reverse-transcribed using the TaqMan miRNA reverse transcription kit (Applied Biosystems, Thermo Fisher Scientific) and miRNA-specific primers (Applied Biosystems, Thermo Fisher Scientific) for hsa-miR-155, hsa-miR-155*, hsa-miR-29b-1*, hsa-miR-150, hsa-miR-146a, hsa-miR-212 and hsa-miR-483-5p. MicroRNA expression levels were then analyzed using the appropriate TaqMan miRNA assay (Applied Biosystems) and TaqMan Universal Master Mix II (Applied Biosystems) according to the manufacturer’s instructions. The ubiquitously expressed U6b snRNA was also quantified as an endogenous control and used to normalize miRNAs expression by using the equation 2^-ΔCt^ or 2^-ΔΔCt^ as needed. The values are mean ± SEM of triplicate determinations.

For the analysis of ATG3, LC3 and GABARAP mRNA levels, total RNA was reverse transcribed as previously described [5] and then analyzed using the appropriate TaqMan assay (Applied Biosystems) and TaqMan Universal Master Mix II (Applied Biosystems) according to the manufacturer’s instructions. Transcript expression was normalized to the GAPDH level by using the equation 2^-ΔCt^; the values are mean ± SEM of triplicate determinations.

Digital PCR experiments were carried out on the BioMark HD System (Fluidigm, San Francisco, CA, USA) by loading hsa-miR-155 or U6b reverse transcribed samples prepared, as described previously, from total RNA, light and heavy phase-associated RNA, into Fluidigm's 37K Digital Array microfluidic chip. Similarly, cDNA samples from total RNA, light and heavy phase-associated RNA, prepared as previously described [5], were used for ATG3 copy number determination by digital PCR.

Fluidigm's 37K Digital Array consists of 48 panels, each of which is further partitioned into 770 reaction chambers. The reaction for each panel was set up with the specific TaqMan miRNA assay probe and with TaqMan Universal Master Mix II (Applied Biosystems) into a final volume of 5 μl. For each sample, six serial dilutions were loaded in triplicate reactions and the chip was then thermocycled and imaged on Fluidigm's BioMark HD real-time PCR system. Positive chambers that originally contained 1 or more molecules has been counted by the Digital PCR analysis software (Fluidigm) and only templates that yielded 150–360 amplified molecules per panel were chosen for technical replication in order to obtain absolute quantification of miRNA copy number.

### Target prediction analysis

For the identification of putative target mRNAs of the selected miRNAs, several prediction algorithms, namely miRanda (http://www.microrna.org) [63], Targetcan (http://www.targetcan.org) [64], Pictar5 (http://pictar.mdc-berlin.de/) [65], DIANA-microT (http://diana.imis.athena-innovation.gr) [66] and miRWalk (http://www.umm.uni-heidelberg.de/apps/zmf/mirwalk) [67] were used.

Only targets consistently present in all five target-prediction databases were taken into consideration for the study.

### Gene ontology analysis

To functionally characterize differentially abundant proteins for biological interpretation, Gene Ontology (GO) analysis was performed. Gene Ontology annotation for the list of putative targets of miR-155, miR-155*, miR-29b-1*, miR-150, miR-146a, miR-212 and miR-483-5p was obtained from miRWalk (http://www.umm.uni-heidelberg.de/apps/zmf/mirwalk). A two-tailed Fisher’s Exact Test with Benjamini-Hochberg multiple testing correction was used to measure the significance of enrichment.

### Gene reporter assay

A 174 bp fragment of 3′-UTR of ATG3 mRNA, containing the seed sequence (GCATTAA) of hsa-miR-155-5p was amplified by PCR (forward primer 5′- GAGAGAGCTCGAAGAGAGCATAAAATCTATCCTAA-3′, reverse primer 5′-GAGACTCGAGTTTTTATTAAACAAGTAAGGCTGG -3′). The fragment was designated as ATG3 3′-UTR and inserted into the pmirGLO Dual-Luciferase miRNA target expression vector (Promega, Madison, WI, USA), between the SacI and XhoI restriction sites.

The hsa-miR-155-5p mimic was used for transient transfection in gain of function experiments. As a negative control, a random sequence miRNA mimic molecule (Ambion, Thermo Fisher Scientific), which has been extensively tested in human cell lines and tissues and validated to not produce identifiable effects on known miRNA function, was employed.

For Dual-Luciferase assay, HEK293T cells (5x10^5^ for each condition) were re-suspended in 100 μl DMEM plus 10% FBS and plated in a 96-well plate.

Lipoplexes were prepared in Opti-MEM serum-free medium (Invitrogen, Thermo Fisher Scientific) by mixing the constructed plasmids together with hsa-miR-155-5p or negative control mimics and Lipofectamine 2000 (1mg/ml) (Invitrogen, Thermo Fisher Scientific). Briefly, 400 ng of the pmirGLO Dual-Luciferase miRNA target expression vector plus 5 pmol of specific miRNA or negative control mimics and 1 μl Lipofectamine 2000 were diluted separately in 25 μl of Opti-MEM medium. The two solutions were gently mixed and incubated for 5 minutes at room temperature and then added to HEK293T cells.

After 24 hours, reporter assay was performed using the kit Dual-Glo Luciferase Assay System (Promega), according to manufacturer’s instructions. Signal of firefly and renilla luciferase was detected using a Victor X2 reader (Perkin-Elmer, Waltham, MA, USA).

### DC transfection

Predesigned miRNA mimic oligonucleotide for hsa-miR-155 was obtained from Ambion (Thermo Fisher Scientific). A negative-control miRNA mimic [control mimic (Ambion, Thermo Fisher Scientific)] was used to address the specificity of the observed effect to the specific miRNA sequence. SiRNA transfection efficiency was determined by transfecting the BLOCK-iT Fluorescent oligo [a FITC-labeled oligonucleotide (Ambion, Life technologies)] together with the specific miRNA or negative control at 1:2 ratio using Lipofectamine RNAiMAX (Invitrogen, Thermo Fisher Scientific). For each condition, 4x10^5^ DC were pleated in a 12-well plate. Lipoplexes were prepared in Opti-MEM serum-free medium (Invitrogen, Thermo Fisher Scientific) at 1:1 ratio. Briefly, miRNA mimics plus fluorescent oligonucleotide and 10 μl of Lipofectamine (1mg/mL) were dilute separately in 250 μl of Opti-MEM medium.

The Lipofectamine solution was added to the miRNA solution and placed for 5 minutes at room temperature and then added to DC. Four hours after incubation, medium was replaced with fresh Opti-MEM medium supplemented with 15% FCS and 2 mM L-glutamine for at least 18 hours. After the indicated time, cells were harvested for transfection efficiency, protein extraction and RNA isolation from twin wells.

Providing that transfection by itself may perturb DC viability, maturation and activation [68], preliminary experiments transfecting an oligo-FITC as control of transfection were performed to monitor DC viability, transfection efficiency as well as expression of the co-stimulatory molecule CD86 by flow cytometry on a FACS Canto (BD Biosciences) (S4 Fig).

This preliminary setting indicated that transfection did not induce cell mortality (S4A Fig). Moreover, around 70–75% of viable culture DC resulted to be transfected and, as expected, CD86 induction was not affected by transfection (S4B Fig and S4C Fig, respectively).

### Immunoblotting analysis

For ATG7, ATG3 and GABARAP determination 10 μg of total protein extract were separated on 10% NuPAGE Bis-Tris gel (Invitrogen, Thermo Fisher Scientific) and electroblotted onto nitrocellulose membranes (GE Healthcare). For LC3 detection 30 μg of total proteins were used for separation on 13.5% NuPAGE Bis-Tris gel and electroblotted onto polyvinylidene fluoride membranes (GE Healthcare Bio-sciences, Pittsburgh, PA, USA). Blots were incubated with primary Abs in 5% nonfat dry milk in TBS plus 0.1% Tween20 (Sigma-Aldrich) overnight at 4°C. Detection was achieved using horseradish peroxidase-conjugate anti-rabbit, anti-mouse or anti-goat (Santa Cruz) secondary Abs and visualized with Clarity Western ECL Substrate (Bio-Rad, Segrate, Italy). ATG7, ATG3 and GABARAP quantifications were performed by calculating their ratio compared to the actin level by using ImageLab software (Bio-Rad). LC3-II/actin ratio was quantified by ImageLab software according to Klionsky et al [69].

### Confocal microscopy

Cells were fixed with 4% paraformaldehyde (Sigma-Aldrich) in PBS followed by permeabilization with 0.2% triton X-100 (Sigma-Aldrich) in PBS. Mycobacteria were stained with Auramine for 20 min and decolorized for 10 min. Cells were then labelled with primary antibody anti-LC3 (Sigma-Aldrich) for 1 hour at room temperature and visualized by means of Cy3-conjugated secondary Ab (Jackson Immunoresearch, Baltimore, PA, USA). For co-localization experiments, after fixation, cells were permeabilized with methanol/acetone (1:1) (Sigma-Aldrich) solution. DC were then labelled with anti-LC3 (Cosmo Bio, Tokio, Japan) and anti-LAMP1 (Abcam, Cambridge, UK) primary antibodies for 1 hour at room temperature and visualized respectively by means of Cy3- and Alexafluor 488-conjugated secondary antibodies (Jackson Immunoresearch).

Coverslips were mounted in SlowFade-Anti-Fade (Invitrogen, Thermo Fisher Scientific) and examined under a confocal microscope (Leica, Nussloch, Germany). Digital images were acquired with Leica software and LC3 dots were quantified with Leica Application Suite X (LAS X) using a set of defined intensity thresholds that were applied to all images. A minimum of 50 cells per sample was counted for triplicate samples per condition in each experiment.

After image acquisition, the co-localization of LC3 and LAMP-1 signals was examined with the ImageJ software. Quantification of co-localization, expressed in terms of Mander's Overlap Coefficient, was calculated using the JacoP plugin of ImageJ software.

### Statistical analysis

Statistical analysis was calculated using a two-tailed Student’s t-test for paired data. A *P* value < 0.05 was considered statistically significant.

## Supporting information

S1 Fig Analysis of miR-146a and miR-155* copy number in total as well as low and high occupancy polysome-associated RNA samples. Total RNA (Total), low occupancy (Light) and high occupancy (Heavy) polysome-associated mRNAs were extracted from untreated DC (CTRL) or DC infected for 16 hours with Mtb.
(A) MiR-155* and (B) miR-146a copy number were determined by digital PCR. Data are represented as the mean copy number per sample ± standard error of the mean of three independent experiments.
(TIF)

S2 Fig Modulation of miR-155 expression at late time points of infection in human DC. Relative expression of miR-155 in human DC left untreated (CTRL) or infected with wild type Mtb at MOI 1, as measured by q-PCR analysis on extracted total RNA samples. All quantification data are normalized to the U6b level using the equation 2^-ΔCt^. The results shown were mean fold change per sample ± standard error of the mean at 8, 24, 32 and 48 hours post-infection on RNA samples derived from a set of experiments independent than those used for microarray (*p = 0.03; **p = 0.02).
(TIF)

S3 Fig Modulation of LC3 protein in human DC treated with LPS or infected with Mtb. LC3 protein levels were determined in human DC left untreated (CTRL), infected with Mtb for 24 hours or treated with LPS (1 μg/ml) by immunoblotting. Actin levels were analyzed to verify the amount of loaded proteins. The results shown are representative of three independent experiments that yielded similar results.
(TIF)

S4 Fig Transfection efficiency of human primary DC. DC were left untreated or transfected for 24 hours with an oligo-FITC and analyzed by flow cytometry. (A) Cell viability was evaluated in DC cultures by staining with the Fixable viability Dye (FvDye). Representative frequencies of live and dead cells are reported in gates. (B) DC transfection efficiency was determined in live-gated cells by means of an oligo-FITC used as assay control during transfection. (C) CD86 surface expression was evaluated in live cells in both un-transfected and oligo-FITC-transfected DC. Mean fluorescence intensity values are shown in each plot.
(TIF)

S1 Table Complete gene ontology analysis on the list of targets for selected miRNAs according to biological process. Functional gene ontology (GO) annotation study for biological process conducted on the list of putative targets obtained for miRNAs de-regulated in DC after Mtb infection, namely miR-155-5p, miR-155-3p, miR-29b-1-5p, miR-150-5p, miR-146a-5p, miR-212-5p and miR483-5p.
(XLSX)

S2 Table Gene ontology analysis on the list of targets for selected miRNAs according to biological process linked to autophagy. Functional gene ontology (GO) annotation study for biological process conducted on the list of putative targets obtained for miRNAs de-regulated in DC after Mtb infection, namely miR-155-5p, miR-155-3p, miR-29b-1-5p, miR-150-5p, miR-146a-5p, miR-212-5p and miR483-5p.
The most significant biological process (BP) correlated with the GO terms ‘autophagy/ autophagic/ lysosome/ endocytosis/ endocytic/ ubiquitin’ are shown in the table. GOBPs linked to autophagy are shown in bold. Only GOBPs showing Fisher’s exact test p-value < 0.05 and Benjamini-Hochberg (BH) corrected p-value < 0.05 were considered for selecting miRNAs involved in autophagy regulation. 'GOBP-Fg', 'GOBP-Bg', 'GenomeFG' and 'GenomeBG' stand for 'number of genes predicted as putative targets in a given GOBP', 'number of genes in a given GOBP', 'total number of target genes within genome' and 'total number of genes within genome'.
(PDF)
