# Supplementary figures and images for: Mycobacterium tuberculosis-induced miR-155 subverts autophagy by targeting ATG3 in human dendritic cells
Source: PLoS Pathog. 2018 Jan 4;14(1):e1006790. doi: 10.1371/journal.ppat.1006790 (PMC5771628; doi:10.1371/journal.ppat.1006790)

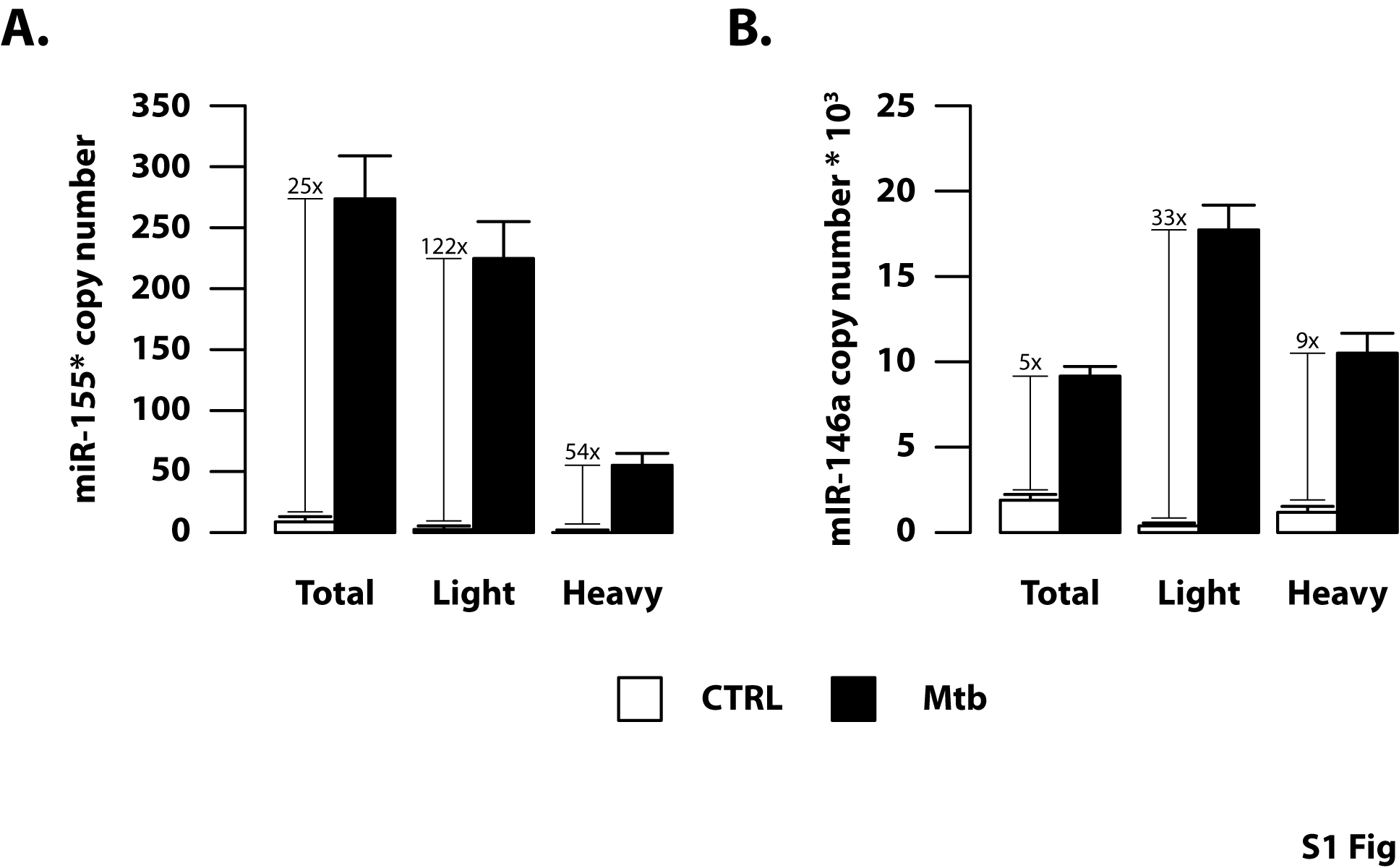

Supplement: S1 Fig — Total RNA (Total), low occupancy (Light) and high occupancy (Heavy) polysome-associated mRNAs were extracted from untreated DC (CTRL) or DC infected for 16 hours with Mtb. (A) MiR-155* and (B) miR-146a copy number were determined by digital PCR. Data are represented as the mean copy number per sample ± standard error of the mean of three independent experiments. (TIF) [file ppat.1006790.s001.tif]

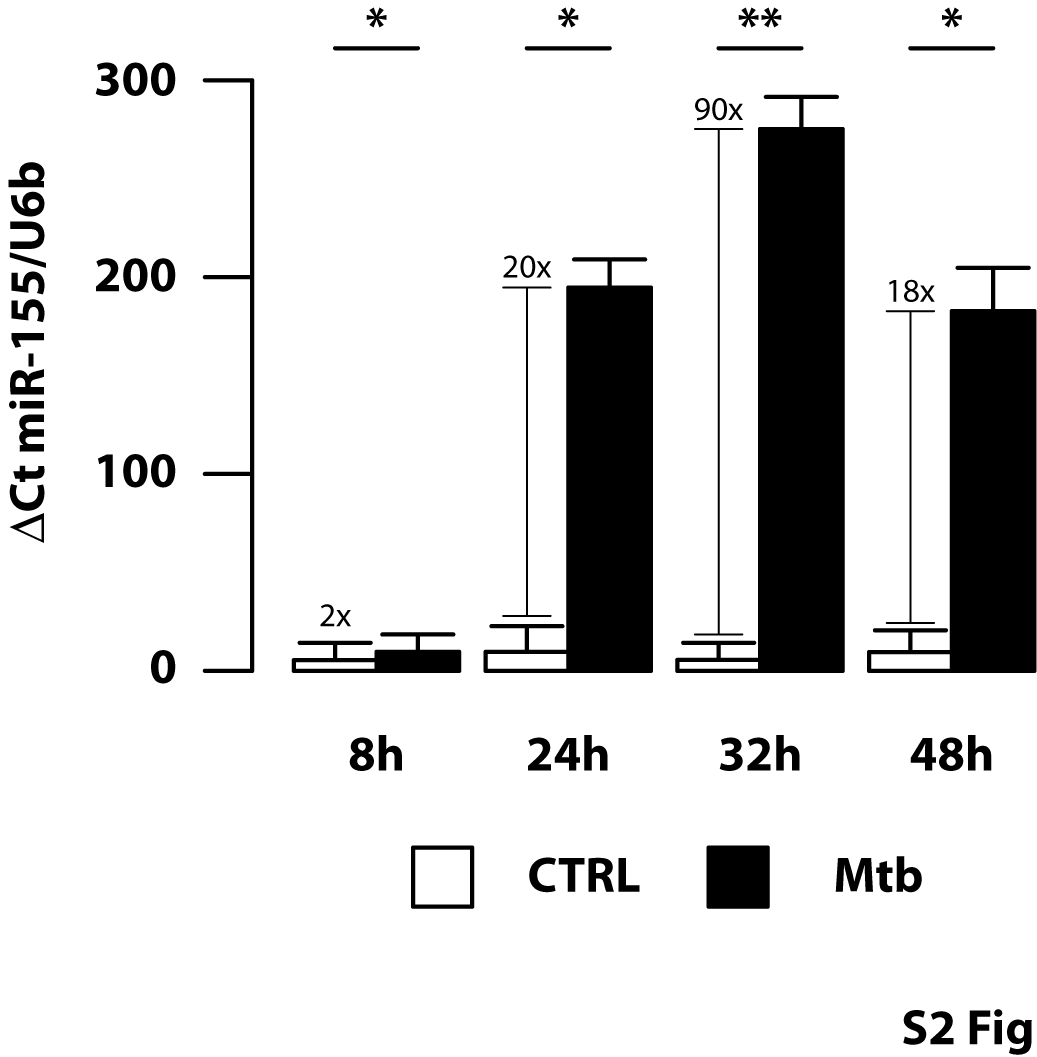

Supplement: S2 Fig — Relative expression of miR-155 in human DC left untreated (CTRL) or infected with wild type Mtb at MOI 1, as measured by q-PCR analysis on extracted total RNA samples. All quantification data are normalized to the U6b level using the equation 2-ΔCt. The results shown were mean fold change per sample ± standard error of the mean at 8, 24, 32 and 48 hours post-infection on RNA samples derived from a set of experiments independent than those used for microarray (*p = 0.03; **p = 0.02). (TIF) [file ppat.1006790.s002.tif]

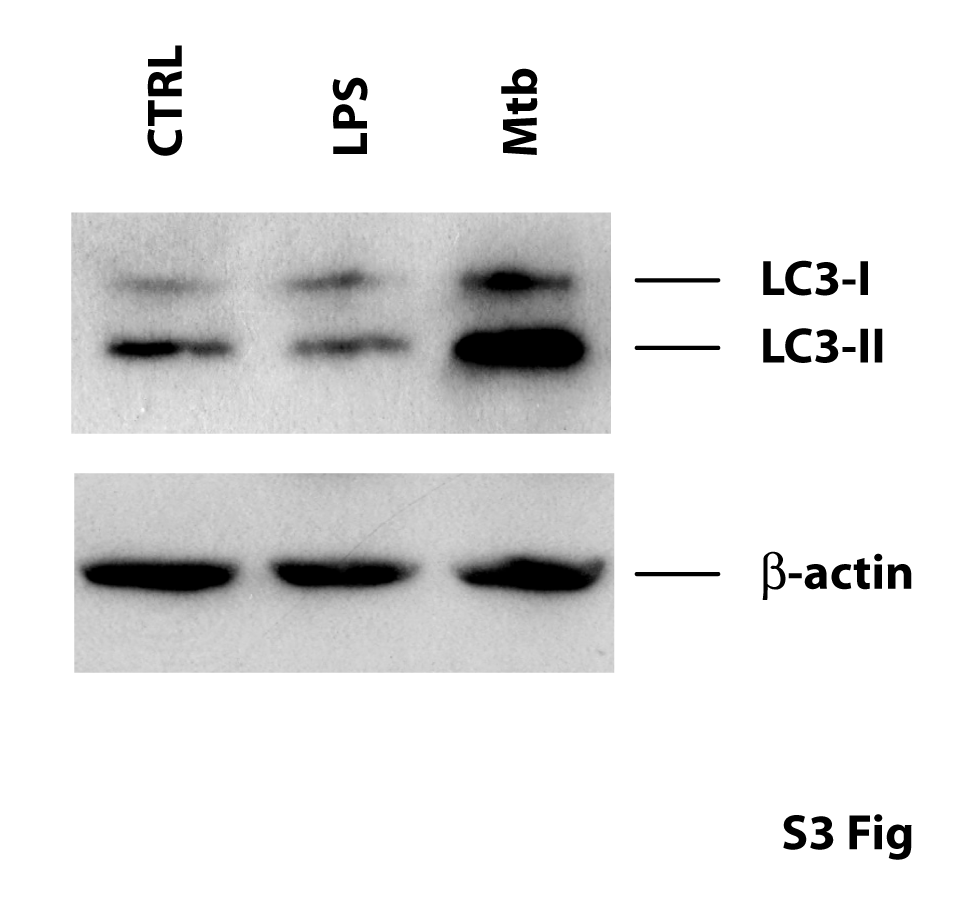

Supplement: S3 Fig — LC3 protein levels were determined in human DC left untreated (CTRL), infected with Mtb for 24 hours or treated with LPS (1 μg/ml) by immunoblotting. Actin levels were analyzed to verify the amount of loaded proteins. The results shown are representative of three independent experiments that yielded similar results. (TIF) [file ppat.1006790.s003.tif]

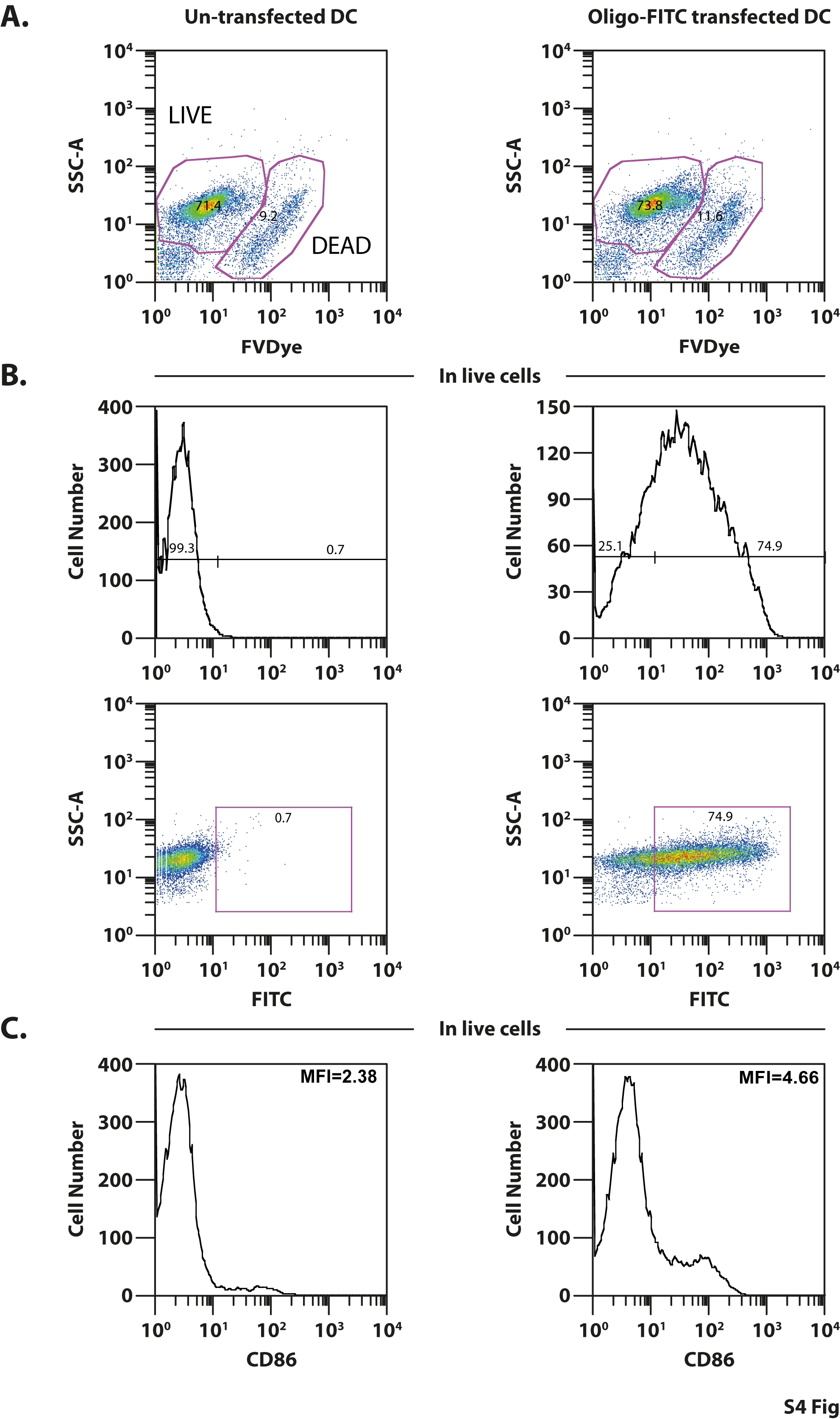

Supplement: S4 Fig — DC were left untreated or transfected for 24 hours with an oligo-FITC and analyzed by flow cytometry. (A) Cell viability was evaluated in DC cultures by staining with the Fixable viability Dye (FvDye). Representative frequencies of live and dead cells are reported in gates. (B) DC transfection efficiency was determined in live-gated cells by means of an oligo-FITC used as assay control during transfection. (C) CD86 surface expression was evaluated in live cells in both un-transfected and oligo-FITC-transfected DC. Mean fluorescence intensity values are shown in each plot. (TIF) [file ppat.1006790.s004.tif]
