# Supplementary material for: Mycobacterium tuberculosis-induced miR-155 subverts autophagy by targeting ATG3 in human dendritic cells
Source: PLoS Pathog. 2018 Jan 4;14(1):e1006790. doi: 10.1371/journal.ppat.1006790 (PMC5771628; doi:10.1371/journal.ppat.1006790)
Supplement: S2 Table — Functional gene ontology (GO) annotation study for biological process conducted on the list of putative targets obtained for miRNAs de-regulated in DC after Mtb infection, namely miR-155-5p, miR-155-3p, miR-29b-1-5p, miR-150-5p, miR-146a-5p, miR-212-5p and miR483-5p. The most significant biological process (BP) correlated with the GO terms ‘autophagy/ autophagic/ lysosome/ endocytosis/ endocytic/ ubiquitin’ are shown in the table. GOBPs linked to autophagy are shown in bold. Only GOBPs showing Fisher’s exact test p-value < 0.05 and Benjamini-Hochberg (BH) corrected p-value < 0.05 were considered for selecting miRNAs involved in autophagy regulation. 'GOBP-Fg', 'GOBP-Bg', 'GenomeFG' and 'GenomeBG' stand for 'number of genes predicted as putative targets in a given GOBP', 'number of genes in a given GOBP', 'total number of target genes within genome' and 'total number of genes within genome'. (PDF) [file ppat.1006790.s006.pdf]

hsa-miR-155	GOBP name	GOBP-Fg	GOBP-Bg	GenomeFG	GenomeBG	Fisher	BH
hsa-miR-155-5p	protein ubiquitination	191	251	12667	19747	2.90E+09	0.0006
hsa-miR-155-5p	protein k48 linked ubiquitination	34	37	12667	19747	0.0001	0.0016
hsa-miR-155-5p	protein ubiquitination involved in ubiquitin dependent protein catabolic process	42	48	12667	19747	0.0002	0.0029
hsa-miR-155-5p	endocytosis	91	116	12667	19747	0.0006	0.0055
hsa-miR-155-5p	ubiquitin dependent protein catabolic process	113	147	12667	19747	0.0006	0.0055
hsa-miR-155-5p	**autophagic vacuole assembly**	20	22	12667	19747	0.004	0.0197
hsa-miR-155-5p	positive regulation of protein ubiquitination	36	44	12667	19747	0.0085	0.0282
hsa-miR-155-5p	protein k63 linked ubiquitination	21	24	12667	19747	0.0106	0.0311
hsa-miR-155-5p	**autophagy**	43	54	12667	19747	0.0104	0.0311
hsa-miR-155-5p	endosome to lysosome transport	21	25	12667	19747	0.0262	0.0436
hsa-miR-155-5p	regulation of endocytosis	15	17	12667	19747	0.0278	0.0436
hsa-miR-155-5p	**positive regulation of autophagy**	11	12	12667	19747	0.0373	0.0507
hsa-miR-155-5p	**autophagic cell death**	7	7	12667	19747	0.0446	0.0522
hsa-miR-155-5p	positive regulation of protein ubiquitination involved in ubiquitin dependent protein catabolic process	7	7	12667	19747	0.0446	0.0522
hsa-miR-155-5p	positive regulation of proteasomal ubiquitin dependent protein catabolic process	24	30	12667	19747	0.0481	0.0537
hsa-miR-155-5p	protein autoubiquitination	26	33	12667	19747	0.0538	0.0549

hsa-miR-155*	GOBP name	GOBP-Fg	GOBP-Bg	GenomeFG	GenomeBG	Fisher	BH
hsa-miR-155-3p	endocytosis	90	116	12140	19747	0.0001	0.0023
hsa-miR-155-3p	protein ubiquitination	178	251	12140	19747	0.0010	0.0079
hsa-miR-155-3p	protein autoubiquitination	28	33	12140	19747	0.0032	0.0168
hsa-miR-155-3p	proteasomal ubiquitin dependent protein catabolic process	41	52	12140	19747	0.0059	0.0224
hsa-miR-155-3p	protein ubiquitination involved in ubiquitin dependent protein catabolic process	38	48	12140	19747	0.0070	0.0248
hsa-miR-155-3p	positive regulation of proteasomal ubiquitin dependent protein catabolic process	25	30	12140	19747	0.0087	0.0273
hsa-miR-155-3p	negative regulation of endocytosis	9	9	12140	19747	0.0125	0.0309
hsa-miR-155-3p	regulation of endocytosis	15	17	12140	19747	0.0166	0.0358
hsa-miR-155-3p	**positive regulation of autophagy**	11	12	12140	19747	0.0247	0.0429
hsa-miR-155-3p	protein deubiquitination	25	32	12140	19747	0.0361	0.0509
hsa-miR-155-3p	**autophagic vacuole assembly**	18	22	12140	19747	0.0361	0.0509
hsa-miR-155-3p	endosome to lysosome transport	20	25	12140	19747	0.0405	0.0540
hsa-miR-155-3p	protein k48 linked ubiquitination	28	37	12140	19747	0.0509	0.0574
hsa-miR-155-3p	protein k63 linked ubiquitination	19	24	12140	19747	0.0539	0.0574

hsa-miR-29b-1*	GOBP name	GOBP-Fg	GOBP-Bg	GenomeFG	GenomeBG	Fisher	BH
hsa-miR-29b-1-5p	protein ubiquitination	242	251	17431	19747	3.77E+07	7.96E+09
hsa-miR-29b-1-5p	endocytosis	112	116	17431	19747	0.0015	0.0073
hsa-miR-29b-1-5p	protein ubiquitination involved in ubiquitin dependent protein catabolic process	48	48	17431	19747	0.0024	0.0105
hsa-miR-29b-1-5p	proteasomal ubiquitin dependent protein catabolic process	51	52	17431	19747	0.0119	0.0304
hsa-miR-29b-1-5p	positive regulation of proteasomal ubiquitin dependent protein catabolic process	30	30	17431	19747	0.0236	0.0419
hsa-miR-29b-1-5p	positive regulation of protein ubiquitination	43	44	17431	19747	0.0281	0.0449
hsa-miR-29b-1-5p	endosome to lysosome transport	25	25	17431	19747	0.0441	0.0566
hsa-miR-29b-1-5p	protein k48 linked ubiquitination	36	37	17431	19747	0.0583	0.0583

hsa-miR-150	GOBP name	GOBP-Fg	GOBP-Bg	GenomeFG	GenomeBG	Fisher	BH
hsa-miR-150-5p	endocytosis	95	116	11961	19747	6.01E+05	2.29E+09
hsa-miR-150-5p	negative regulation of protein ubiquitination	18	20	11961	19747	0.0041	0.0164
hsa-miR-150-5p	protein ubiquitination	170	251	11961	19747	0.0109	0.0268
hsa-miR-150-5p	regulation of endocytosis	15	17	11961	19747	0.0138	0.0294
hsa-miR-150-5p	**positive regulation of macroautophagy**	9	10	11961	19747	0.0498	0.0525
hsa-miR-150-5p	receptor mediated endocytosis	35	48	11961	19747	0.0518	0.0530

hsa-miR-146a	GOBP name	GOBP-Fg	GOBP-Bg	GenomeFG	GenomeBG	Fisher	BH
hsa-miR-146a-5p	protein ubiquitination	210	251	13936	19747	9.95E+07	3.18E+09
hsa-miR-146a-5p	protein ubiquitination involved in ubiquitin dependent protein catabolic process	46	48	13936	19747	1.16E+09	0.0002
hsa-miR-146a-5p	protein k48 linked ubiquitination	35	37	13936	19747	0.0003	0.0028
hsa-miR-146a-5p	endocytosis	96	116	13936	19747	0.0018	0.0086
hsa-miR-146a-5p	ubiquitin dependent protein catabolic process	117	147	13936	19747	0.0085	0.0219
hsa-miR-146a-5p	**autophagy**	46	54	13936	19747	0.0101	0.0253
hsa-miR-146a-5p	endosome to lysosome transport	23	25	13936	19747	0.0104	0.0254
hsa-miR-146a-5p	**positive regulation of autophagy**	12	12	13936	19747	0.0152	0.0306
hsa-miR-146a-5p	protein targeting to lysosome	10	10	13936	19747	0.0306	0.0419
hsa-miR-146a-5p	endocytic recycling	10	10	13936	19747	0.0306	0.0419
hsa-miR-146a-5p	**positive regulation of macroautophagy**	10	10	13936	19747	0.0306	0.0419
hsa-miR-146a-5p	proteasomal ubiquitin dependent protein catabolic process	43	52	13936	19747	0.0339	0.0453
hsa-miR-146a-5p	lysosome organization	22	25	13936	19747	0.0377	0.0489
hsa-miR-146a-5p	protein k63 linked ubiquitination	21	24	13936	19747	0.0478	0.0523

hsa-miR-212	GOBP name	GOBP-Fg	GOBP-Bg	GenomeFG	GenomeBG	Fisher	BH
hsa-miR-212-5p	protein ubiquitination	229	251	16309	19747	6.65E+09	0.0011
hsa-miR-212-5p	positive regulation of protein ubiquitination	43	44	16309	19747	0.0022	0.0110
hsa-miR-212-5p	endocytosis	107	116	16309	19747	0.0022	0.0110
hsa-miR-212-5p	protein ubiquitination involved in ubiquitin dependent protein catabolic process	46	48	16309	19747	0.0062	0.0216
hsa-miR-212-5p	protein k48 linked ubiquitination	36	37	16309	19747	0.0073	0.0231
hsa-miR-212-5p	protein k63 linked ubiquitination	24	24	16309	19747	0.0101	0.0268
hsa-miR-212-5p	protein autoubiquitination	32	33	16309	19747	0.0143	0.0302
hsa-miR-212-5p	positive regulation of proteasomal ubiquitin dependent protein catabolic process	29	30	16309	19747	0.0235	0.0392
hsa-miR-212-5p	receptor mediated endocytosis	45	48	16309	19747	0.0228	0.0392
hsa-miR-212-5p	proteasomal ubiquitin dependent protein catabolic process	48	52	16309	19747	0.0387	0.0510
hsa-miR-212-5p	lysosome organization	24	25	16309	19747	0.0524	0.0579
hsa-miR-212-5p	scf dependent proteasomal ubiquitin dependent protein catabolic process	15	15	16309	19747	0.0566	0.0579
hsa-miR-212-5p	endosome to lysosome transport	24	25	16309	19747	0.0524	0.0579

hsa-miR-483-5p	GOBP name	GOBP-Fg	GOBP-Bg	GenomeFG	GenomeBG	Fisher	BH
hsa-miR-483-5p	protein ubiquitination	220	251	15246	19747	1.72E+09	0.0002
hsa-miR-483-5p	endocytosis	105	116	15246	19747	0.0001	0.0014
hsa-miR-483-5p	proteasomal ubiquitin dependent protein catabolic process	50	52	15246	19747	0.0001	0.0016
hsa-miR-483-5p	protein ubiquitination involved in ubiquitin dependent protein catabolic process	46	48	15246	19747	0.0004	0.0029
hsa-miR-483-5p	ubiquitin dependent protein catabolic process	128	147	15246	19747	0.0017	0.0084
hsa-miR-483-5p	protein k48 linked ubiquitination	35	37	15246	19747	0.0048	0.0178
hsa-miR-483-5p	positive regulation of receptor mediated endocytosis	13	13	15246	19747	0.0345	0.0518
hsa-miR-483-5p	protein autoubiquitination	30	33	15246	19747	0.0385	0.0537
hsa-miR-483-5p	negative regulation of protein ubiquitination	19	20	15246	19747	0.0390	0.0537
hsa-miR-483-5p	**positive regulation of autophagy**	12	12	15246	19747	0.0448	0.0563
hsa-miR-483-5p	protein k48 linked deubiquitination	12	12	15246	19747	0.0448	0.0563
hsa-miR-483-5p	endosome to lysosome transport	23	25	15246	19747	0.0535	0.0599
hsa-miR-483-5p	protein monoubiquitination	17	18	15246	19747	0.0599	0.0599
